# Notch signaling pathway: architecture, disease, and therapeutics

**DOI:** 10.1038/s41392-022-00934-y

**Published:** 2022-03-24

**Authors:** Binghan Zhou, Wanling Lin, Yaling Long, Yunkai Yang, Huan Zhang, Kongming Wu, Qian Chu

**Affiliations:** Department of Oncology, Tongji Hospital, Tongji Medical College, Huazhong University of Science and Technology, 430030 Wuhan, China

**Keywords:** Drug development, Target identification, Cancer microenvironment, Differentiation, Neurogenesis

## Abstract

The NOTCH gene was identified approximately 110 years ago. Classical studies have revealed that NOTCH signaling is an evolutionarily conserved pathway. NOTCH receptors undergo three cleavages and translocate into the nucleus to regulate the transcription of target genes. NOTCH signaling deeply participates in the development and homeostasis of multiple tissues and organs, the aberration of which results in cancerous and noncancerous diseases. However, recent studies indicate that the outcomes of NOTCH signaling are changeable and highly dependent on context. In terms of cancers, NOTCH signaling can both promote and inhibit tumor development in various types of cancer. The overall performance of NOTCH-targeted therapies in clinical trials has failed to meet expectations. Additionally, *NOTCH* mutation has been proposed as a predictive biomarker for immune checkpoint blockade therapy in many cancers. Collectively, the NOTCH pathway needs to be integrally assessed with new perspectives to inspire discoveries and applications. In this review, we focus on both classical and the latest findings related to NOTCH signaling to illustrate the history, architecture, regulatory mechanisms, contributions to physiological development, related diseases, and therapeutic applications of the NOTCH pathway. The contributions of NOTCH signaling to the tumor immune microenvironment and cancer immunotherapy are also highlighted. We hope this review will help not only beginners but also experts to systematically and thoroughly understand the NOTCH signaling pathway.

## Introduction

The *NOTCH* gene was first named in studies of *Drosophila melanogaster* with notched wings in the 1910s^1–3^. Homologs of NOTCH were then identified in multiple metazoans, and all these NOTCH homologs shared similar structures and signaling components^4–7^. NOTCH variants were also found in ancient humans and were found to be involved in brain size control^8^. Generally, NOTCH is considered an ancient and highly conserved signaling pathway. NOTCH signaling participates in various biological processes across species, such as organ formation, tissue function, and tissue repair; thus, aberrant NOTCH signaling may cause pathological consequences.

In the past two decades, various drugs targeting NOTCH signaling have been tested in preclinical and clinical settings, yet no drug has been approved. Recent studies indicate that the NOTCH pathway is far more extensive and complicated than previously believed. As immunotherapy has revolutionized cancer treatment, NOTCH signaling and its relation with antitumor immunity have attracted the attention of scientists.

This review aims to illustrate the history, architecture, regulatory mechanisms, relation to health and diseases, and therapeutic applications of the NOTCH signaling pathway. In regard to specific behaviors of the NOTCH signaling pathway, we tried to focus on studies of mammals rather than those of other animals. We hope this review will help not only beginners but also experts to systematically and thoroughly understand the NOTCH signaling pathway.

## A brief history of notch signaling

The *NOTCH* gene was first described in a study of *D. melanogaster* mutants with notched wings in the 1910s^1,3,4^. Haploinsufficiency of *NOTCH* caused *D. melanogaster* to have notches at the end of their wings, while complete insufficiency was lethal. The discovery of this phenotype inspired the later proposed nomenclature. The *D. melanogaster NOTCH* gene was then isolated^9^ and sequenced^10^ in the 1980s, and the putative NOTCH protein was found to span the membrane and contain many epidermal growth factor (EGF)-like repeats^11^. Studies of NOTCH signaling in *D. melanogaster* then increased^12–18^, drawing attention to the whole signaling pathway. In 1988 and 1989, LIN-12 and GLP-1 were identified as NOTCH homologs in *Caenorhabditis elegans*^4,5^, seemingly associated with *C. elegans* development^5,19,20^. In 1990, XOTCH (a homolog of *D. melanogaster* NOTCH) was identified in Xenopus^6^, and the cDNA of the mammalian *NOTCH* gene was cloned^7^. Since then, research on NOTCH in other animals has gained popularity. More details of NOTCH signaling have been clarified, and as such, NOTCH has been recognized as an ancient and highly conserved signaling pathway across metazoans^21–26^.

**Fig. 1:**
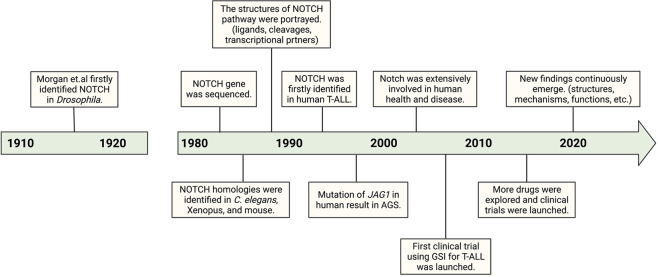
A brief history of the NOTCH signaling pathway. T-ALL, T cell acute lymphoblastic leukemia; AGS, Alagille syndrome; GSI, γ-secretase inhibitor

In 1991, the NOTCH gene was first linked to human T cell acute lymphoblastic leukemia (T-ALL). In 1997, Alagille syndrome (AGS) was found to be caused by the mutation of JAG1, which encodes a ligand of NOTCH1^27,28^. AGS is a noncancerous autosomal dominant disorder characterized by the abnormal development of multiple organs. Since these discoveries, the relationship of NOTCH with human health and diseases has been extensively studied. In addition, translational studies have been performed. The first clinical trial involving NOTCH signaling was launched in 2006, using a γ-secretase inhibitor to treat patients with T-ALL or other leukemias^29,30^. It was halted due to severe diarrhea, yet the results largely promoted the therapeutic targeting of NOTCH signaling. Various drugs and antibodies targeting other components of NOTCH signaling have been explored in preclinical and clinical settings, although none has yet been approved. In recent years, many new studies have been appearing, such as detailed structural analyses^31–33^, analyses of complicated regulatory mechanisms^34,35^, and analyses of diversified functions in health and diseases^36–38^, highlighting some unexplored areas of NOTCH signaling. A brief history of NOTCH signaling is shown in Fig. 1. A strong understanding of NOTCH signaling is required; thus, more efforts are needed.

## The architecture of notch signaling

**Fig. 2:**
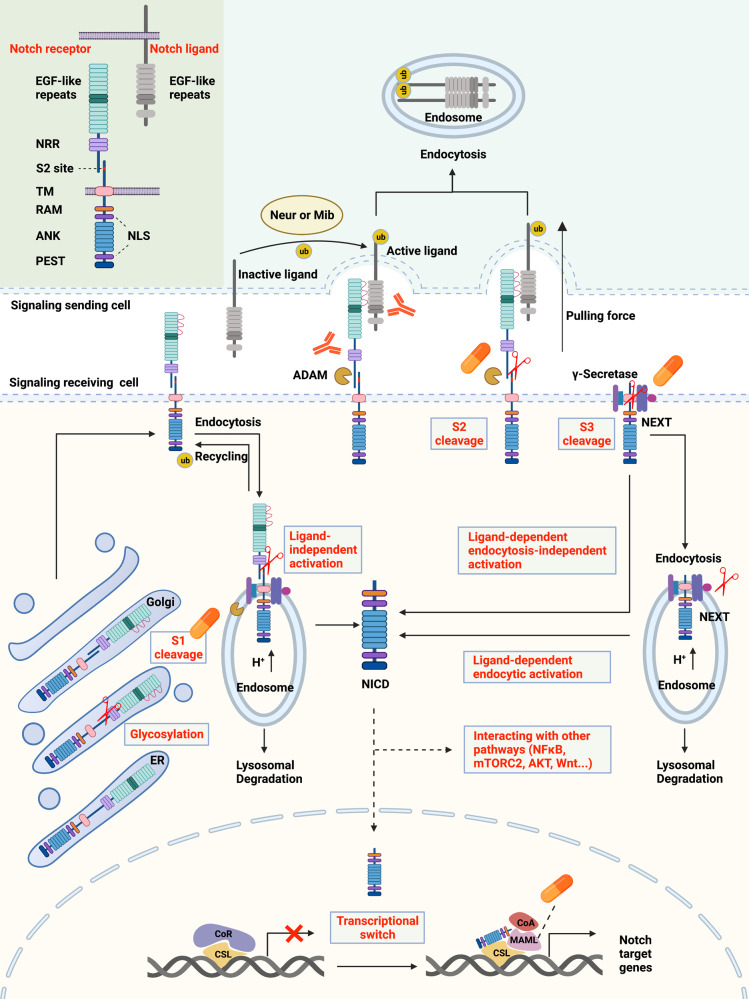
Overview of the NOTCH signaling pathway and therapeutic targets. In signal-receiving cells, NOTCH receptors are first generated in the ER and then trafficked to the Golgi apparatus. During trafficking, NOTCH receptors are glycosylated at the EGF-like repeat domain (red curves). Then, in the Golgi apparatus, NOTCH receptors are cleaved into heterodimers (S1 cleavage) and transported to the cell membrane. With the help of ubiquitin ligases, some of the NOTCH receptors on the cell membrane are endocytosed into endosomes. Endosomes contain an acidic environment with ADAMs and γ-secretase. The NOTCH receptors in endosomes can be recycled to the cell membrane, cleaved into NICD, or transported into lysosomes for degradation. In signal-sending cells, NOTCH ligands are distributed on the cell membrane and can bind to NOTCH receptors on signal-receiving cells. However, the ligands are inactive before ubiquitylation by Neur or Mib. After ubiquitylation, ligands can be endocytosed, thus producing a pulling force for the binding receptors. Without the pulling force, the S2 site (red marks) of NOTCH receptors is hidden by the NRR domain, and thus, the NOTCH receptors are resistant to cleavage by ADAMs. With the pulling force, the NRR domain is extended, therefore exposing the S2 site for cleavage. ADAMs and the pulling force are both necessary for S2 cleavage. After S2 cleavage, the remaining part of the NOTCH receptor is called NEXT. NEXT can be further cleaved on the cell membrane by γ-secretase or endocytosed into endosomes. In the former mode, NICD is released on the cell membrane. In the latter mode, NEXT can be cleaved into NICD or transported into lysosomes for degradation. In total, there are three approaches to generate NICD, classified as ligand-independent activation, ligand-dependent endocytosis-independent activation, and ligand-dependent endocytic activation. NICD can be translocated into the nucleus or remain in the cytoplasm to crosstalk with other signaling pathways, such as NFκB, mTORC2, AKT, and Wnt. The classical model proposes that, in the absence of NICD, CSL binds with corepressors to inhibit the transcription of target genes. Once NICD enters the nucleus, it can bind with CSL and recruit MAMLs, releasing corepressors, recruiting coactivators, and thus promoting the transcription of NOTCH target genes. There are two main approaches to inhibit NOTCH signaling for therapy. One is designing inhibitors of the key components of the pathways, including the enzymes that participate in S1 cleavage, ADAMs, γ-secretase, and MAML. The other one is producing antibody-drug conjugates against NOTCH receptors and ligands. The protein structures of NOTCH ligands and receptors are shown in the top left corner. NICD, NOTCH intracellular domain; ADAM, a disintegrin and metalloproteinase domain-containing protein; Neur, Neuralized; Mib, Mindbomb; NRR, negative regulatory region; NEXT, NOTCH extracellular truncation; CSL, CBF-1/suppressor of hairless/Lag1; MAMLs, Mastermind-like proteins; TM, transmembrane domain; RAM, RBPJ association module; ANK, ankyrin repeats; PEST, proline/glutamic acid/serine/threonine-rich motifs; NLS, nuclear localization sequence; CoR, corepressor; CoA, coactivator; ub, ubiquitin

The NOTCH signaling pathway has certain characteristics. Classical signaling pathways, mediated by G protein-coupled receptors (GPCRs)^39^ and enzyme-linked receptors^40^, have multiple intermediates between the membranous receptors and nuclear effectors. However, the canonical NOTCH signaling pathway has no intermediate, with receptors directly translocated into the nucleus after three cleavages^21,41,42^ (Fig. 2). In addition, S2 cleavage of NOTCH receptors is triggered by interactions with ligands expressed on adjacent cells, indicating a rather narrow range of NOTCH signaling. NOTCH signaling is involved in multiple aspects of metazoans’ life^42^, including cell fate decisions, embryo and tissue development, tissue functions and repair, as well as noncancerous and cancerous diseases. Thus, understanding of the architecture of the NOTCH signaling pathway is necessary.

### The receptors and ligands of NOTCH signaling

*D. melanogaster* has only one NOTCH receptor^9^. *C. elegans* has two redundant NOTCH receptors, LIN-12 and GLP-1^4^. Mammals have four NOTCH paralogs, NOTCH1, NOTCH2, NOTCH3, and NOTCH4^21^, showing both redundant and unique functions. In humans, NOTCH1, NOTCH2, NOTCH3, and NOTCH4 are located on chromosomes 9, 1, 19, and 6, respectively. After transcription and translation, NOTCH precursors are generated in the endoplasmic reticulum (ER) and then translocated into the Golgi apparatus. In the ER, the NOTCH precursors are initially glycosylated at the EGF-like repeat domain. Glycosylations include O-fucosylation, O-glucosylation, and O-GlcNAcylation, which are catalyzed by the enzymes POFUT1, POGLUT1, and EOGT1, respectively^43^. Subsequently, in the Golgi apparatus, O-fucose is extended by the Fringe family of GlcNAc transferases, while O-glucose is extended by the xylosyltransferases GXYLT1/2 and XXYLT1^44–46^. The glycosylation of NOTCH is vital to its stability and function. Alteration of core glycosylation enzymes severely inhibits the activity of NOTCH signaling^47–51^, making these enzymes vital for further research.

The glycosylated NOTCH precursors undergo S1 cleavage in the Golgi apparatus before being transported to the cell membrane. The cleavage always occurs at a conserved site (heterodimerization domain) and is catalyzed by a furin-like protease, cutting NOTCH into a heterodimer (mature form). Here, we take mouse NOTCH1 as an example to illustrate the structure of mature NOTCH on the cell membrane.

The extracellular domain (N-terminal) contains 36 EGF-like repeats and a negative regulatory region (NRR)^43^. The 11th and 12th EGF-like repeats usually interact with ligands^43^, although a new study found that many more motifs of the extracellular domain are involved in ligand binding^52^. The NRR domain is composed of three cysteine-rich Lin12-NOTCH repeats (LNRs) and a heterodimerization region critical for S2 cleavage. Located after the membrane-spanning region, the intracellular RBPJ association module (RAM) domain is responsible for interacting with transcription factors in the nucleus, and seven ankyrin repeat (ANK) domains are observed in the RAM domain. Nuclear localization sequences are located on both sides of the ANK domains. At the end of the intracellular domain (C-terminus), there are conserved proline/glutamic acid/serine/threonine-rich motifs (PEST domains) that contain degradation signals and are thus critical for the stability of the NOTCH intracellular domain (NICD). Mammalian NOTCH2-4 have similar structures to NOTCH1, diverging mainly in the number of EGF-like repeats, the glycosylation level of the EGF-like repeats, and the length of the PEST domains. The level of NOTCH receptors on the cell membrane is controlled by constitutive endocytosis, which is promoted by ubiquitin ligases. An appreciable amount of NOTCH receptors are ubiquitinated and degraded in the proteosome, while the rest are expressed on the cell membrane to transmit signals.

Humans and mice have five acknowledged NOTCH ligands^21,53,54^: delta-like ligand 1 (DLL1), delta-like ligand 3 (DLL3), delta-like ligand 4 (DLL4), Jagged-1 (JAG1), and Jagged-2 (JAG2), all of which present redundant and unique functions. For instance, DLL1 governs cell differentiation and cell-to-cell communication^54^, DLL3 suppresses cell growth by inducing apoptosis^55^, DLL4 activates NF-κΒ signaling to enhance vascular endothelial factor (VEGF) secretion and tumor metastasis^56^, JAG1 enhances angiogenesis^54^, and JAG2 promotes cell survival and proliferation^54^.

The structures of the NOTCH ligands are partially similar to those of the receptors. The ligands are also transmembrane proteins, and the extracellular domains contain multiple EGF-like repeats, which determine the crosstalk with corresponding receptors. The levels and functions of the ligands are also controlled by ubiquitylation and endocytosis (discussed in the section “Ligand ubiquitylation”).

### The canonical NOTCH signaling pathway

The mature NOTCH receptors on the cell membrane are heterodimers, with the heterodimerization domain being cleaved in the Golgi apparatus (S1 cleavage). Generally, binding to extracellular domains of NOTCH receptors allows ligands to initiate endocytosis. Such endocytosis induces receptors to change their conformation, exposing the enzymatic site for S2 cleavage^57^. Receptors then experience S3 cleavage, changing into the effector form: NOTCH intracellular domain (NICD). NICD is degraded in the cytoplasm or transported into the nucleus to regulate the transcription of target genes (Fig. 2).

S2 cleavage is the only ligand-binding step and is thus vital for signal initiation. The structural basis of S2 cleavage is illustrated in Fig. 2. The S2 site (metalloprotease site) is hidden by the LNR domain in the silent phase, referred to as the “autoinhibited conformation”^58^. Once bound with ligands, the receptor extends the LNR domain and exposes the S2 site for cleavage^59–61^. The core enzymes for S2 cleavage include a disintegrin and metalloprotease 10 (ADAM 10) and its isoforms ADAM 17 and ADAMTS1^62–64^, which are popular targets for drug discovery. The product of S2 cleavage (larger part) is composed of the transmembrane domain and the intracellular domain, which is also called NOTCH extracellular truncation (NEXT)^65^.

NEXT is further cleaved at the S3 site, releasing NICD, which can be translocated into the nucleus and function as a transcription factor. The enzyme responsible for S3 cleavage is γ-secretase, which contains the catalytic subunits presenilin1 or presenilin2 (PS1 or PS2)^66,67^, APH-1, PEN-2, and nicastrin (NCT)^68^. However, the classical substrates for γ-secretase contain NOTCH receptors and amyloid precursor protein (APP), the successive cleavage of which is related to Alzheimer’s disease^69–72^. The structural basis for γ-secretase to recognize NOTCH or APP had remained unclear until recently, when Yigong Shi’s team elucidated the structural basis^31,32^. In short, the transmembrane helix of NOTCH or APP closely interacts with the surrounding transmembrane helix of PS1 (the catalytic subunit of γ-secretase); thus, the hybrid β-sheet promotes substrate cleavages, although some differences exist between NOTCH and APP^73^. Structural information would accelerate the discovery of substrate-specific inhibitors of NOTCH and APP. Additionally, S3 cleavage can occur both on the cell membrane and in the endosome after NEXT is endocytosed, termed the endocytosis-independent model and endocytic-activation model, respectively^74^.

After release from the cell membrane, NICD is translocated into the nucleus to regulate gene transcription, the mechanism of which may be related to the nuclear localization sequences of NICD and importins alpha 3, 4, and 7^75^. However, the details of this translocation remain unclear. CBF-1/suppressor of hairless/Lag1 (CSL, also called recombination signal binding protein-J, RBPJ) is a ubiquitous transcription factor (TF) that recruits other co-TFs to regulate gene expression^76,77^. The target genes of NOTCH signaling are largely determined by the Su (H) motif of CSL, which is responsible for DNA binding^21^. The canonical NOTCH target gene families are Hairy/Enhancer of Split (HES) and Hairy/Enhancer of Split related to YRPW motif (HEY)^21^.

In the traditional model of NICD regulating gene transcription^21,42,78,79^, CSL recruits corepressor proteins and histone deacetylases (HDACs) to repress the transcription of target genes without NICD binding. NICD binding can change the conformation of the CSL-repressing complex, dissociating repressive proteins and recruiting activating partners to promote the transcription of target genes. The transcriptional coactivator Mastermind-like protein (MAML) is one of the core activating partners that can recognize the NICD/CSL interface, after which it recruits other activating partners. Drugs targeting MAML are under study.

Recently, Kimble et al. used single-molecule fluorescence in situ hybridization to study the NOTCH transcriptional program in germline stem cells of *C. elegans* and found that NICD dictated the probability of transcriptional firing and thus the number of nascent transcripts^80^. However, NICD did not orchestrate a synchronous transcriptional response in the nucleus, in contrast to that seen in the classical model. Gomez-Lamarca et al. found similar results in *D. melanogaster*^81^. NICD promoted the opening of chromatin and enhanced the recruitment of both the NICD-containing activating CSL complex and the NICD-free repressive CSL complex. Bray et al. proposed a new model to interpret their findings. In the NOTCH-off state, chromatin is compact, and only the NICD-free (repressing) CSL complex regulates transcription. In the NOTCH-on state, chromatin is loosened and bound to both NICD-containing (activating) and NICD-free (repressive) CSL complexes. Because the number of activating complexes is greater than that of repressive complexes after NICD enters the nucleus, NICD promotes the transcription of target genes. Bray et al. further reported that nucleosome turnover occurred frequently at NOTCH-responsive regions and depended on the Brahma SWI/SNF chromatin remodeling complex^82^. Consistently, Kimble et al. found that NOTCH signaling regulated the duration of the transcriptional burst but not the intensity of signaling or the time between bursts^83^. Oncogenic NOTCH is also considered to enhance repositioning to promote the transcription of genes, such as MYC^84^. In general, the new model from Bray et al. helps explain the flexibility of NOTCH signaling, although the details still require further elucidation.

### The noncanonical NOTCH signaling pathway

Pathways other than canonical signaling pathway are also able to initiate signaling, classified as noncanonical NOTCH signaling pathways. Although the mature NOTCH receptors on the cell membrane are capable of ligand binding, some are endocytosed for renewal. Endocytosed NOTCH receptors can return to the cell membrane, be degraded in lysosomes or activated in endosomes (ligand-independent activation)^74,85^. Interestingly, endosome trafficking can also be regulated by NOTCH signaling^86^. Endosomes have been proven to contain ADAM and γ-secretase^87^. Ligand-independent activation of NOTCH signaling is vital to T cell development^88^. One example of ligand-independent activation is T cell receptor (TCR)-mediated self-amplification^87^. The activated TCR/CD3 complex can activate the signaling axis of LCK-ZAP70-PLCγ-PKC. PKC then activates ADAM and γ-secretase on the endosome to initiate S2 and S3 cleavage and thus NOTCH signaling. Activated NOTCH signaling can further upregulate immune-related genes to amplify the immune response.

Independent of CSL, NICD can interact with the NF-κB, mTORC, PTEN, AKT, Wnt, Hippo, or TGF-β pathways at the cytoplasmic and/or nuclear level to regulate the transcription of target genes^34,89–96^. The crosstalk between NICD and NF-κB affects the malignant properties of cervical cancer^89^, colorectal cancer^97^, breast cancer^98^, and small-cell lung cancer cells^99^. Targeting the NF-κB pathway could be an effective way to block noncanonical NOTCH signaling.

In addition to those mentioned above, there is a newly identified mechanism of noncanonical activation. In the classical model, S3 cleavage is necessary for NOTCH receptors to release NICD and thus regulate the transcription of target genes. However, membrane-tethered NOTCH may activate the PI3K-AKT pathway, promoting the transcription of interleukin-10 and interleukin-12^100^. In blood flow-mediated NOTCH signaling, the transmembrane domain instead of NICD recruits other partners to promote the formation of an endothelial barrier^35^. NOTCH3 itself can promote the apoptosis of tumor endothelial cells, independent of cleavage and transcription regulation^101^. The JAG1 intracellular domain can promote tumor growth and epithelial–mesenchymal transition (EMT) without binding to NOTCH receptors^102^. These noncanonical mechanisms provide this ancient signaling pathway with more unique functions while massively increasing its complexity.

### The mechanisms regulating NOTCH signaling

#### Glycosylation

The glycosylation of NOTCH receptors on specific EGF-like repeats is crucial for the maturation of receptors, which also affects signaling output. First, O-fucosylation catalyzes the enzyme Pofut1 to affect ligand binding. Elimination of Pofut1 greatly influences the ligand binding of NOTCH signaling in embryonic stem cells, lymphoid cells, and angiogenic cells of mice^103–105^. The aberration of fringe family proteins, which catalyzes the elongation of O-fucose, can also affect ligand binding^106–109^. Second, O-glucose of NOTCH receptors is involved in S2 cleavage. Alteration of O-glucosylation damages the proteolysis of NOTCH receptors after ligand binding^110,111^. Third, the sites of O-glycosylation, such as EGF 12, are important regions for ligand binding, the loss of which decreases NOTCH signaling in T cells^112^. Furthermore, EGF 28 might contribute to DLL1-mediated NOTCH1 signaling^113^. Targeting glycosylation is also thought to effectively inhibit NOTCH signaling^114^.

#### Receptor trafficking

After S1 cleavage, most mature NOTCH proteins are transported to the cell membrane. However, reaching the membrane does not guarantee stability. NOTCH receptors are constitutively endocytosed through a process modulated by ubiquitin ligases such as FBXW, NUMB, ASB, DTX1, NEDD4, ITCH, and CBL^74,115–118^. Endocytosed NOTCH can be recycled to the cell membrane or trapped in the cytoplasm^74^; thus, receptor trafficking can directly affect the level of NOTCH receptors on the cell membrane. Furthermore, the endocytosed NOTCH receptors in the cytoplasm can be degraded or activated. Degradation is usually initiated by the endosomal sorting complex required for transport (ESCRT) system^119–122^, the failure of which also lays the foundation for receptor activation. However, the mechanism of ligand-independent activation remains clear^123–125^. The balance between degradation and activation after endocytosis is closely related to downstream signaling^79^. The specific distribution of receptors and ligands on the cell membrane can also influence the regional intensity of NOTCH signaling^79^.

#### Ligand ubiquitylation

Unlike the ubiquitylation of NOTCH receptors, ubiquitylation of ligands (usually catalyzed by Neuralized (Neur) and Mindbomb (Mib)) in signal-sending cells is necessary for signaling activation. Without Neur or Mib, NOTCH signaling decreases significantly^126–128^. One explanation is that the endocytosis of ligands promotes exposure of the NRR domain of the receptor for S2 cleavage^129,130^.

#### Cis-inhibition

Receptors and ligands expressed on different cells can initiate signal transduction. However, receptors and ligands expressed on the same cell both inhibit and activate the whole signaling pathway, termed cis-inhibition and cis-activation^79,131^. DLL3 seems to operate only in cis-inhibition^132,133^. The loss of DLL3 increases NOTCH activity during T cell development in vivo^133^. DLL1-NOTCH1 can function in both cis- and trans-activation^131^. Thus, the balance between cis- and trans-interactions can be vital to signaling output.

#### Other regulatory mechanisms

Various signals regulate the transcription of NOTCH receptors and thus the whole signaling pathway, such as AKT, RUNX1, SIRT6, CBFB, and DEC1^134–138^. Many noncoding RNAs regulate the level of NOTCH receptors, such as microRNA-26a, microRNA-26b, microRNA-153, microRNA-182, and microRNA-34a^139–142^. Nitric oxide regulates the activity of ADAM17 and USP9X and ultimately NOTCH signaling^143,144^. Calzado et al. found that dual-specificity tyrosine-regulated kinase 2 (DYRK2) phosphorylated the NOTCH1 intracellular domain to promote its degradation by FBXW7^145^. In the classical model, NOTCH signaling is prompted through the interaction between receptors and ligands in extracellular domains. However Suckling et al. found that the interaction between the C2 domain of NOTCH ligands and the phospholipid membrane of receptor-containing cells modulated NOTCH signaling.^146^ This finding provides a possible explanation for the diversified consequences of NOTCH signaling mediated by different ligand–receptor interactions.

## Notch signaling in organ development and repair

**Fig. 3:**
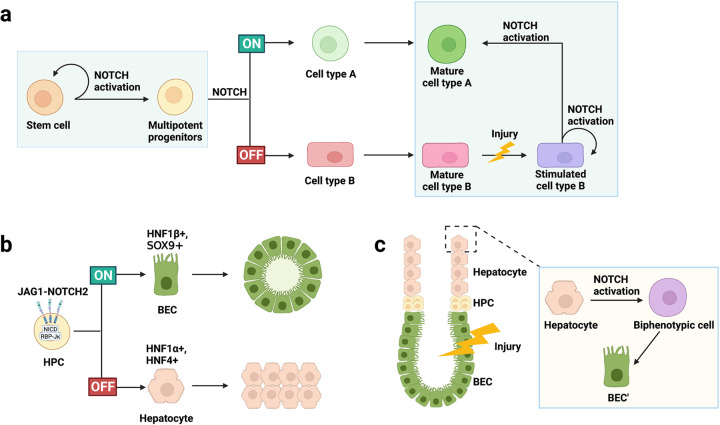
The role of NOTCH signaling in body development and damage repair. NOTCH signaling is involved in regulating the differentiation and function of stem cells, affecting organ production and damage repair. **a** NOTCH signaling promotes the self-renewal of stem cells, induces multipotent progenitors for lineage selection, and generates different terminal cells; when the organ is damaged, cell type A is damaged and destroyed, and the stimulated cell type B rapidly upregulates the expression of NOTCH signaling to promote their own proliferation, and is partially redifferentiated into cell type A. **b** Highly activated NOTCH induces the expression of bile duct cell-enriched transcription factors and promotes the differentiation of multipotent hepatocyte progenitors into bile duct epithelial cells. **c** In liver injury, BEC are damaged and destroyed. NOTCH signaling is highly expressed in hepatocytes, which are further transformed into biphenotypic cells, which manifests the biliary tract morphology, and finally generate new BEC (BEC’) to form small tubular structures. HPC, hematopoietic progenitor cell; BEC, bile duct epithelial cell; SOX9, SRY-related high-mobility group box 9; HNF, hepatocyte nuclear factor

As a highly conserved signaling pathway, NOTCH deficiency leads to serious embryonic lethality. NOTCH signaling is active in the early stage of embryonic development but is maintained at a low level in the mature stage of body development. It also increases rapidly under conditions of injury or stress and is indispensable for development and injury repair (Fig. 3). First, NOTCH signaling promotes the self-renewal and dedifferentiation of stem and progenitor cells, thus maintains progenitor stemness and the stem cell pool. Among these cells, neural stem cells^147–149^ and multipotent progenitor cells (MPCs)^150,151^ are classic representatives. Different combinations of NOTCH ligands and receptors promote stem cell proliferation and inhibit terminal differentiation. Second, NOTCH signaling is involved in the selection of cell fate. Based on temporal and spatial expression of NOTCH ligands, receptors, and cell-enriched transcription factors, NOTCH signaling induces fixed differentiation of progenitor cells, such as differentiation of cardiac progenitor cells into endocardial cells and hepatoblasts into bile duct lineage cells^152,153^. Furthermore, NOTCH signaling is vital to maintaining the homeostasis of the body in normal regeneration and damage repair. NOTCH signaling can rapidly regulate the dynamic transformation of cells to maintain physiological homeostasis, such as stem cells and tail cells in angiogenesis, through lateral inhibition^154–157^. It also induces the differentiation and transformation of mature cells to promote damage repair, for example, in liver regeneration^158^. Last, numerous ligands and receptors are involved in NOTCH signaling and have specified temporal and spatial expression in various organs and tissues, although the consequences are similar.

### NOTCH and somitogenesis

The somitogenesis of vertebrates occurs in a strict order and is regulated by the segmentation clock. It is closely related to the expression of oscillating genes regulated by NOTCH, Wnt and FGF signaling^159–162^. NOTCH signaling triggers an excitatory signal, causing presomitic mesoderm (PSM) to transition into a self-sustaining cyclic oscillation state^163,164^. The gene oscillation period is consistent with the half-life of HES7^165^ and induces lunatic fringe (*Lfng*) transcription. LFNG, as a glycosyl transferase that can modify the extracellular domain of NOTCH after translation and periodically blocks the cleavage of NOTCH receptors, causes the formation of cyclic NICD^166–168^. PSM is a group of self-sustaining oscillating cells, but the synchronous oscillation between depends on the transmission of NOTCH signaling^169–171^. LFNG inhibits the activation of NOTCH signaling in neighboring cells by regulating the function of DLL1^164,172,173^. In *Lfng*-knockout mice, PMS oscillation fails to synchronize, but PMS oscillation amplitude and period remain unaffected^170^. This finding further demonstrates that LFNG is a key coupling factor for synchronous oscillations between cells.

### NOTCH and skeleton

In the growth and development of MPC, NOTCH signaling regulates and inhibits the production of osteoblasts^151^, chondrocytes^174–178^, and osteoclasts^179,180^ through different ligands and receptors (NOTCH1, NOTCH2, JAG1, DLL1) as well as the downstream target gene (SRY-related high-mobility group box 9, *SOX9*). In addition, the latest research shows that inhibiting glucose metabolism can guide NOTCH to regulate MPC^150^, proving the complex role of NOTCH signaling in the skeletal microenvironment. In the mouse model, the absence of NOTCH signaling leads to depletion of MPC and nonunion of fractures^181^, consistent with the finding that activated JAG1-NOTCH signaling reduces MPC senescence and cell cycle arrest. Interestingly, using γ-secretase inhibitors intermittently and temporarily for fractures significantly promotes cartilage and bone callus formation, as well as superior strength^182^. This indicates that NOTCH signaling exerts its function in a temporally and spatially dependent manner.

### NOTCH and cardiomyogenesis

During heart wall formation, NOTCH signaling regulates the ratio of cardiomyocytes to noncardiomyocytes by inhibiting myogenesis, further promoting atrioventricular canal remodeling and maturation, EMT development and heart valve formation^183–185^. In the endocardium layer, the DLL4-NOTCH1-mediated *Hey1/2*-*Bmp2*-*Tbx2* signaling axis is a complex negative feedback regulation loop, where overexpressed *Tbx2* can in turn inhibit upstream *Hey* expression^186–189^. In embryos lacking key NOTCH signaling molecules such as *Notch1*, *Rbpj*, *Hey1*/*Heyl,* or *Hey2*, EMT development is hindered, and endocardial cells are activated but fail to scatter and invade heart glia^190^. NOTCH signaling affects the expression of the cadherin 5^190^ and the TGF-β family member bone morphogenetic protein 2 (BMP2)^186,189^. In addition, by downregulating VEGFR2, a key negative regulator of EMT within atrioventricular canals (AVCs), NOTCH signaling further induces EMT. Studies have found that active NOTCH1 is most highly expressed in endocardial cells at the base of the trabecular membrane. Bone morphogenetic protein 10 (BMP10)^191^ and Neuregulin 1 (NRG1)^192^ are key molecules of NOTCH signaling that regulate the proliferation, differentiation, and correct folding of cardiomyocytes during trabecular development.

### NOTCH and the vasculature

NOTCH4 and DLL4 are specifically expressed on vascular endothelial cells (ECs)^184,193^. Deficiencies in NOTCH signaling result in serious defects in the vasculature of the embryo and yolk sac during embryonic development^194^ as well as abnormal development of multiple organs, such as the retinal vasculature^195,196^ and uterine blood vessels^197^ in rats. At the cellular level, the vascular system mainly includes ECs, pericytes and vascular smooth muscle cells (VSMCs). Under stressors such as hypoxia, resting ECs quickly transform into a state of active growth and high plasticity and then dynamically transform between tip cells (TCs) and stalk cells (SCs) through lateral inhibition rather than direct lineage changes^154,155^. This cascade reaction between DLL4-mediated NOTCH signaling and VEGFA-VEGFR2 signaling induces ECs near dominant TCs to maintain a high level of NOTCH signaling, inhibiting their differentiation into TCs^198,199^. NOTCH signaling activates the Wnt pathway through feedback regulation to maintain the connection between ECs, promoting vascular stability^200^. In addition, DLL4-NOTCH can maintain arterial blood–retinal barrier homeostasis by inhibiting transcytosis^201^. NOTCH signaling is also important for the development of VSMCs^202,203^. Blocking Notch signaling in neural crest cells, especially NOTCH2 and NOTCH3, results in vascular dysplasia, aortic defects, and even bleeding^202,204–206^. The regulation of the downstream transcription factors *PAX1*, *SCX,* and *SOX9* by NOTCH signaling is vital for regulating the differentiation of progenitor cells in the sclera toward VSMCs^207^.

NOTCH signaling acts decisively in the arteriovenous differentiation of endothelial cells^208,209^. NOTCH signaling induces the expression of the arterial marker ephrin B2 and inhibits that of the venous marker EphB4, thereby regulating the number and diameter of arteriovenous vessels^210,211^. In mice with dysfunctional mutations of NOTCH signaling molecules such as *Notch1*, *Dll4, Hey1,* or *Hey2*, the arterial subregion is defective, while venous differentiation is hyperactive, leading to unexpected bleeding^210,212,213^. Before blood perfusion, active NOTCH signaling on the arterial side can be detected. High levels of VEGF, ERK/MAP kinase and Wnt pathway components increase DLL4 expression^214,215^, and the transcription factors *Fox1C* and *Fox2C* promote DLL4 activation^216^. Interestingly, ECs can sense and respond to laminar flow through NOTCH1, similar to the shear stress response, transforming the hemodynamic mechanical force into an intracellular signal, which is necessary for vascular balance^217,218^.

### NOTCH and the hemopoietic system

NOTCH signaling is important in the differentiation, development, and function of hematopoietic system cells, both lymphocytes and myeloid cells. In early embryonic development, the hematopoietic endothelium forms hematopoietic stem cells through NOTCH-dependent endothelial-to-hematopoietic transition^219^. NOTCH signaling is fundamental in maintaining the number and stemness of hematopoietic stem cells^220^. In lymphocyte development, the absence of NOTCH1 or CSL in early hematopoietic progenitor cells (HPCs) leads to thymic T cell development retardation and B cell accumulation, with HES1 being the key mediator^221^. Naïve thymocytes highly express NOTCH and immediately downregulate NOTCH1 expression once they successfully pass β-selection. Some scholars propose that NOTCH-mediated T cell development is initiated in the prethymic niche^222,223^. For example, bone mesenchymal cells outside the thymus can cross-link with HPCs through NOTCH ligands on the surface to promote the generation of T cell lineages^224,225^. Shreya S et al. induced the production of HSPC-derived CD7+ progenitor T cells with DLL4 and VCAM-1 in vitro engineering, and these cells further differentiated into mature T cells after thymus transplantation^226^. Regarding B cells, the development of splenic marginal zone B (MZB) cells depends on DLL1-NOTCH2 signaling^227,228^. In addition, it was found that active NOTCH2 signaling can mediate the lineage conversion of follicular B cells into MZB cells so that mature B cell subpopulations can quickly and dynamically transform based on the needs of the immune system^229,230^. The development of innate lymphoid cells (ILCs) was recently found to be NOTCH-dependent^231–233^, and the response of different subtypes of ILCs to NOTCH signaling is heterogeneous^234,235^. It is interesting that ILCs can activate MZB cells through DLL1 to enhance antibody production^236^. Regarding myeloid cells, NOTCH signaling is significant in the development of macrophages^237,238^, dendritic cells^239,240^, granulocytes^241^, etc.

### NOTCH and the liver

NOTCH signaling plays a key role in determining the fate of biliary tract cells and directing the correct morphogenesis of the biliary tree. Active NOTCH signaling, especially mediated by NOTCH2 and JAG1, promotes the expression of transcription factors enriched in bile duct cells, induces the differentiation of hepatocytes toward bile duct cells, and promotes the formation of the bile duct plates^152,153^. The expression of SOX9, a downstream molecule of NOTCH signaling, is synchronized with the asymmetric development of the bile duct^152,242^, with a mouse model of liver-specific deletion of *Sox9* echoing this finding. Interestingly, delayed biliary tract development caused by liver-specific deletion of *Sox9* eventually resolves in a spontaneous manner, proving that SOX9 plays a major role in timing regulation through the development of the biliary tract^243^.

The liver has a strong compensatory regeneration ability, where NOTCH signaling responds quickly with significant upregulation, and the transformation of hepatocytes into bile duct-like cells can be observed (Fig. 3c). Similarly, high levels of dual-phenotype hepatocytes can also be observed in liver slices of patients with early liver diseases. Additionally, in a mouse orthotopic liver transplantation model, a high level of NOTCH1 (NICD and HES1) signaling was found to have a protective effect on hepatocytes during ischemia–reperfusion injury, regulating macrophage immunity^244^. In incomplete liver injury, NOTCH signaling mediates the proliferation and differentiation of facultative progenitor cells, thereby promoting biliary tract repair. Such damage repair can be induced mainly by NOTCH2^245,246^, consistent with the discovery of the role of NOTCH2 signaling in the differentiation and selection of liver progenitor cells during liver development.

### NOTCH and the gastrointestinal tract

Studies have shown that NOTCH signaling prevents embryonic epithelial cells from differentiating into secretory lineages^247^, with *Hes1* being the main negative regulator^248^. Highly activated NOTCH signaling promotes the differentiation of intestinal stem cells toward intestinal epithelial cells^249^. Inhibiting NOTCH signaling increases the differentiation of secretory goblet cells^250^. Additionally, the lateral inhibition of NOTCH/DLL1 and the synergy of the Wnt signaling pathway^250^ drive Paneth cell differentiation and subsequent crypt formation^251^. NOTCH signaling is also essential in the lineage selection of gastric stem cells^252^ and necessary to maintain the homeostasis of gastric antral stem cells^253^. Activated NOTCH signaling in differentiated mature gastric epithelial cells induces their dedifferentiation^254^. NOTCH signaling is also vital to the proliferation of pancreatic progenitor cells and their correct differentiation into mature pancreatic cells^255,256^. DLL1 and DLL4 are specifically expressed in β cells, while JAG1 is expressed in α cells^257^. The DLL1-NOTCH-HES1 signaling axis promotes the growth and fate selection of multipotent pancreatic progenitor cells, while JAG1 competes with DLL1 to induce opposite effects^258^.

### NOTCH and the nervous system

NOTCH signaling negatively regulates neurogenic phenotypes^259–262^. Its absence induces differentiation of neural stem cells toward neurons at the cost of glial cell production, in both *D. melanogaster* and vertebrates^263–266^. There are two mainstream models: the classic lateral inhibition model that is similar to vascular development^267^ and the model involving oscillatory expression of HES1, NEUROG2 and DLL1^268^. In addition, NOTCH signaling promotes the differentiation of most glial cell subtypes, except for oligodendrocytes. In the peripheral nervous system, the interaction between NOTCH signaling and Hairy2 is vital for the development of neural crest cells, although the specific regulatory mechanism remains unclear^269^. Active NOTCH signaling blocks the occurrence and stratification of the trigeminal nerve, leading to disorders of brain development. Furthermore, NOTCH signaling drives intestinal neural crest cells to develop into precocious glial cells in Hirschsprung disease^270,271^. These results indicate that NOTCH signaling participates in neural crest differentiation, but further exploration is required^272^.

### NOTCH and other organs or systems

NOTCH signaling functions throughout lung development and the damage repair process^273^. Components of NOTCH signaling are highly expressed in various cells and tissues during lung development. Inhibition of NOTCH signaling or RBPJ deficiency causes defects in proximal airway differentiation, club-cell secretion inhibition, and excessive proliferation of ciliated cells and neuroendocrine cells. NOTCH2 is the main factor activating alveolar morphogenesis and maintaining airway epithelial integrity^274^. NOTCH signaling mediates the balance between the proliferation and differentiation of basal cells^275^. In damage repair, NOTCH2 in basal cells is activated, promoting the separation of cell lineages and producing secretory cells^276^.

NOTCH signaling is important in cell lineage selection, epidermal homeostasis and skin function^277^. NOTCH signaling in the skin promotes cell differentiation^278^, while NOTCH in hair follicles inhibits cell differentiation, promotes proliferation and maintains stemness. Notch signaling is also closely related to cilia cell proliferation, differentiation and morphogenesis and may be involved in asymmetric cell division in the embryonic epidermis^279,280^. NOTCH signaling regulates sebaceous gland stem cells directly and indirectly. In *Rbpj*-deficient mice, the differentiation of sebaceous stem cells is inhibited, and the number of sebaceous glands (SGs) is reduced, with compensatory, enlarged SGs still existing^281^. Many skin diseases have been found to have NOTCH signaling changes, such as hidradenitis suppurativa, psoriasis, and atopic dermatitis^282,283^.

## Notch signaling in noncancerous diseases

As mentioned above, NOTCH signaling is essential for body development and homeostasis, indicating that NOTCH signaling is vital for the occurrence and development of diseases. Most genetic diseases caused by NOTCH mutations have a low incidence and lack effective treatment. For example, the first discovered related disorder, Cerebral autosomal dominant arteriopathy with subcortical infarcts and leukoencephalopathy (CADASIL), has no effective treatment other than supportive treatment. The prognosis of only a few patients with AGS can be improved through liver transplantation, suggesting that further research is necessary. Most of the diseases caused by nonmutant NOTCH signaling abnormalities present corresponding developmental characteristics. New and interesting findings have appeared recently. For example, NOTCH signaling may be related to alcohol-associated preference, playing an important role in nonalcoholic fatty liver disease. We will now focus on the manifestations of NOTCH signaling abnormalities in diseases caused by congenital or nongenetic mutations (Table 1).

**Table 1:** NOTCH Signaling in Noncancerous diseases

Disease type	Key NOTCH components	Affected organs/tissue	Main manifestations	Ref.
*Diseases related to abnormal expression of NOTCH signaling factors caused by gene mutation*				
CADASIL	NOTCH3	Arterioles of the brain	Particulate osmophilic substances are deposited near VSMCs; arterial damage and brain damage	^285,286,291,636^
Alagille syndrome	NOTCH2, JAG1	Multiple organs and systems	Absence of bile ducts, cholestasis; peripheral arterial stenosis; specific facial features	^28,293,301^
Spondylocostal dysostosis	DLL3, MESP2, HES7	Vertebral column	Malformed ribs, asymmetrical rib cage, short trunk	^306,637^
Hajdu-Cheney disease	NOTCH2	Skeletal tissue	Truncated NOTCH2 proteins escape ubiquitylation and degradation, mediating active NOTCH2 signaling; osteoporosis, craniofacial anomalies	^638–640^
Left ventricle cardiomyopathy	MIB1	Heart	Promotes the engulfment of NOTCH ligands, inhibits NOTCH signal transduction; hinders ventricular myocardium development	^641,642^
Adams-Oliver syndrome	NOTCH1, RBPJ, DLL4	Skin, limbs	Scalp hypoplasia, terminal transverse limb defects	^643,644^
Bicuspid aortic valve disease	NOTCH1, RBPJ, JAG1	Cardiac valves	Related to valvular disorders of EMT and valve calcification	^645–647^
Schizophrenia	NOTCH4	Brain	One of the strongest candidate susceptibility genes for schizophrenia	^648,649^
*Diseases related to abnormal expression of NOTCH signaling factors caused by factors other than gene mutation*				
Pulmonary arterial hypertension	NOTCH1, NOTCH3	Pulmonary vasculature	ECs and VSMCs hyperproliferation and activation; vascular remodeling, pulmonary artery obstruction	^331,332,650,651^
Nonalcoholic steatohepatitis	NOTCH1, JAG1	Liver	Abnormal NOTCH signaling activation in liver cells promotes osteopontin expression and secretion	^315,316,318^
Osteoarthritis	RBPJ, JAG1, HES1	Articular cartilage	Abnormally high expression of NOTCH factors in OA; NOTCH signaling plays a dual regulatory role, participating in both damage repair and progression of disease, with temporal and spatial specificity	^320–323^
Graft versus host disease	NOTCH1, NOTCH2, JAG1, DLL1, DLL4	Immune system	Activation and promotion the differentiation and function of T cells; increases the BCR responsiveness of patient B cells	^337,339,340,652^
Pancreatitis	NOTCH1, JAG1, HES1	Pancreas	Associated with tissue regeneration and renewal after pancreatitis; contributes to the differentiation and proliferation of acinar cells	^653–655^
Multiple sclerosis	JAG1	Myelin sheath	Inhibition of oligodendrocyte maturation and differentiation and formation of the myelin sheath	^656–658^
Duchenne muscular dystrophy	JAG1	Skeletal muscle	Associated with the depletion and senescence of MPCs	^659,660^
Klippel-Feil syndrome	RIPPLY2	Vertebra	Regulates the asymmetric development of embryos	^661,662^
Alcohol associative preference	NOTCH/Su(H)	Neurons	Affects alcohol-related neuroplasticity in adults	^663^

*CADASIL* Cerebral autosomal dominant arteriopathy with subcortical infarcts and leukoencephalopathy, *VSMCs* vascular smooth muscle cells, *MESP2* mesoderm posterior 2, *MIB1* mindbomb homolog 1, *RBPJ* recombination signal binding protein-J, *EMT* epithelial–mesenchymal transition, *ECs* endothelial cells, *OA* osteoarthritis, *BCR* B-cell receptor, *MPCs* multipotent progenitor cells, *RIPPLY2* ripply transcriptional repressor 2, *Su(H)* suppressor of hairless

### Diseases associated with abnormal expression of NOTCH signaling related to mutations

#### CADASIL

CADASIL syndrome, an arteriolar vascular disease mediated by dominant mutations in the NOTCH3 gene, is the most common hereditary cause of stroke and vascular dementia in adults^284,285^. NOTCH3 is mainly expressed in VSMCs and pericytes, especially arterioles. In a study of 50 unrelated CADASIL patients, 45 with *NOTCH3* pathogenic mutations^286^ presented abnormal folding of NOTCH3 and deposition of osmophilic particles near VSMCs^287,288^, and cerebral arteries showed reduced lumen diameter unassociated with chronic hypertension^289^. *Notch3*-knockout mice show obvious structural abnormalities of arterioles and loss of vascular smooth muscle, simulating some CADASIL vascular changes, but are insufficient to constitute a complete CADASIL pathological model^290^. Attempts have been made to simulate the main pathological features of CADASIL regarding vascular damage and unique brain damage^291^, such as introducing *Notch3* pathogenic point mutations into large P1-derived artificial chromosomes (PACs) to construct transgenic mouse models with large genome fragments of *Nothc3* pathogenic mutations^292^ and using patient-derived induced pluripotent stem cell modeling. Evidently, NOTCH3 is pathogenic when mutated, although its underlying mechanism remains unclear.

#### Alagille syndrome

AGS is an autosomal dominant genetic disease caused by abnormal NOTCH signaling, with *JAG1* mutations being predominant (greater than 90%) and *NOTCH2* mutations being second most common (5%)^27,28,293^. AGS affects multiple organs throughout the body, inducing, for example, abnormal development of the liver, heart, vasculature, bones, eyes, and maxillofacial dysplasia. Liver damage is the most prominent and is characterized by a lack of interlobular bile ducts and varying degrees of cholestasis, jaundice, and itching. AGS is one of the most important causes of chronic cholestasis in children. Symptoms ameliorate with age, yet there is still no effective treatment other than liver transplantation^294,295^. These findings are consistent with the roles of JAG1 and NOTCH2 in bile duct development and morphological maintenance mentioned above. Interestingly, according to statistics, *JAG1* has more than 430 mutation sites outside of mutation hotspots. Similarly, its phenotype is highly variable, and a correlation between genotype and phenotype has not yet been found^296–299^. Thus, it remains a mystery how changes in different NOTCH receptors and ligands affect the occurrence and development of AGS. There was no research model with the characteristics of AGS until the structural defect model of the biliary tree using biopsies from AGS patients was developed, and experiments have indicated that AGS liver organoids may be a good human 3D model of AGS^300^. *JAG1* homozygous mutations often lead to embryonic lethality in mice. Andersson et al. successfully constructed mice homozygous for a missense mutation (H268Q) in *Jag1* (*Jag1*Ndr/Ndr), and these mice showed a decreased rate of embryonic lethality and recapitulation of all AGS features. Surviving mice presented with the classic absence of bile ducts and other features of AGS, including defects of the heart, vasculature, and eyes^301,302^. In the pathological tissues of patients and mouse models, Joshua et al. found that the expression level of SOX9 was negatively correlated with the severity of AGS liver damage, and overexpression of SOX9 could rescue bile duct loss in Jag1+/– mouse models. One explanation is that overexpressed SOX9 can be recruited to the NOTCH2 promoter to upregulate the expression of NOTCH2 in the liver, thereby compensating for the decreased expression of the JAG1 ligand^303^. These new research models and related experimental data have promoted and informed further research on AGS.

#### Congenital scoliosis

Sporadic and familial congenital scoliosis (CS) refers to the lateral curvature of at least one spine segment caused by fetal spinal dysplasia. Studies have shown that CS is closely related to genetic factors, environmental factors, developmental abnormalities, and NOTCH signaling^304^. Several key NOTCH genes involved in the segmentation clock mechanism may explain the features of a genetic model of a rare syndrome characterized mainly by CS-spondylocostal dysostosis (SCD)^305,306^.

When analyzing genes in the families of SCD patients, multiple mutation sites in DLL3 are found, and the phenotype of pyramidal dysplasia in *Dll3*-free mice is similar to that of SCD patients^307^. The genetic correlation between *DLL3* mutation and spinal rib dysplasia has been reported^308^, and *DLL3* deletion alone is unable to induce a complete SCD phenotype. In addition, *Mesp2* is a downstream gene of NOTCH in somite differentiation, and abnormal expression of its 4 pairs of base repeats are closely related to SCD. *Mesp2*-knockout mice have spinal chondrodysplasia and serve as the current main research model^309,310^. In mice, inactivation of *Lfng* or *Hes7* can distort the development of the spine and ribs, with corresponding mutations also found in patients^311,312^. Furthermore, environmental damage to genetically susceptible mice affects the penetrance and severity of the CS phenotype, especially under hypoxic conditions, providing an explanation for the family phenotypic variation of SCD^313^.

### Diseases associated with abnormal expression of NOTCH signaling not related to mutations

#### Nonalcoholic steatohepatitis

There is almost no NOTCH activity in hepatocytes of healthy adults, while NOTCH activity is slightly elevated in hepatocytes of people with simple steatosis and highly elevated in the hepatocytes of nonalcoholic steatohepatitis (NASH)/fibrosis patients; NOTCH activity is positively correlated with the severity of the disease. In NASH patients or high-fat diet-induced NASH mouse models, the expression of NOTCH1, NOTCH2, and HES1 is highly elevated, which activates neoadipogenesis and increases liver steatosis^314–316^. Such abnormal NOTCH activation may mainly be induced by JAG1/NOTCH signaling triggered by intercellular TLR4^317^. NOTCH-active hepatocytes can upregulate the expression of SPP1 through the downstream transcription factor *SOX9*, promoting secretion of osteopontin (OPN) by hepatocytes and activating hepatic stellate cells (HSCs) to induce liver fibrosis^318^.

#### Osteoarthritis

The expression level of NOTCH signaling components is low in the articular cartilage of healthy adults but higher in osteoarthritis (OA) biopsies^319,320^. After trauma, NOTCH signaling is abnormally activated in joint tissues, and its continuous activation can cause early and progressive OA-like lesions. However, transient NOTCH signaling activation helps synthesize cartilage matrix and promotes joint repair^321^. Inhibition of NOTCH signaling was found to significantly reduce the proliferation of OA chondrocytes. However, the specific inhibition of cartilage NOTCH signaling and the decrease in MMP13 abundance in the joint can delay cartilage degeneration^322^. Eventually, long-term loss of NOTCH signaling will cause cartilage homeostasis imbalance and bone destruction. The findings above suggest that *Rbpj* and *Hes1* play a major mediating role^323^. In summary, NOTCH signaling presents duality when regulating the physiology and pathology of articular cartilage, and its effects are depending on temporal and spatial factors.

#### Lung-related diseases

Allergic asthma is mainly driven by the Th2 immune response, where NOTCH signaling activates the expression of the key transcription factor Gata3^324,325^. Preclinical studies of γ-secretase inhibitor (GSI) have also proven that inhibiting NOTCH signaling reduces the asthma phenotype^326,327^. NOTCH signaling plays an important role in promoting Th2 cell lymph node regression and lung migration^328^. NOTCH4 has been further proven to be vital in the occurrence of asthma. Repeated exposure to allergens can induce regulatory T cells (Tregs) to upregulate the expression of NOTCH4, dampening their immunoregulatory function and activating downstream Wnt and Hippo pathways. These factors turn Tregs into Th2 and Th17 cells, maintaining persistent allergic asthma^95,329^. In addition, upregulation of JAG1 expression is found in lung tissues of patients with interstitial pulmonary fibrosis. In chronic lung injury, repeated injury promotes continuous upregulation of JAG1 by inhibiting CXCR7, leading to the continuous activation of NOTCH in surrounding fibroblasts and inducing profibrotic responses^330^. NOTCH3 is an important mediator of pulmonary artery remodeling in pulmonary arterial hypertension (PAH) that mediates the excessive proliferation and dedifferentiation of VSMCs^329^. In addition, the regulation of NOTCH1 in endothelial cells also promotes the progression of PAH^331,332^. Chronic obstructive pulmonary disease (COPD) is a common lung disease associated with smoking. Studies have shown that smoking and PM2.5 exposure promote the activation of NOTCH signaling, leading to the imbalance of T cell subsets and immune disorders, thus aggravating COPD^333–335^.

#### Other diseases

NOTCH signaling is a regulator of the CD4+ T cells that cause graft versus host disease (GVHD)^336^. Inhibition of NOTCH signaling reduces target organ injury and germinal center formation, significantly reducing the severity and mortality of GVHD^337,338^. Activated NOTCH signaling can directly activate reactive T cells and promote their function^339^. The responsiveness of patients’ B cell receptors is also significantly enhanced by activated NOTCH signaling^340^. NOTCH signaling is also involved in regulating the glomerular filtration barrier. Abnormal activation of NOTCH1 signaling in the glomerular endothelium inhibits the expression of VE-cadherin and induces albuminuria through the transcription factors Snai1 and Erg^36^. In adult pancreatic β cells, the abnormal activation of NOTCH signaling, especially DLL1 and DLL4, can promote β cell proliferation. A large number of naïve, dysfunctional β-cells, which proliferate but are unable to secrete insulin normally, causes glucose intolerance^257,341^.

## Notch signaling in cancers

### NOTCH as an oncogene in cancers

**Fig. 4:**
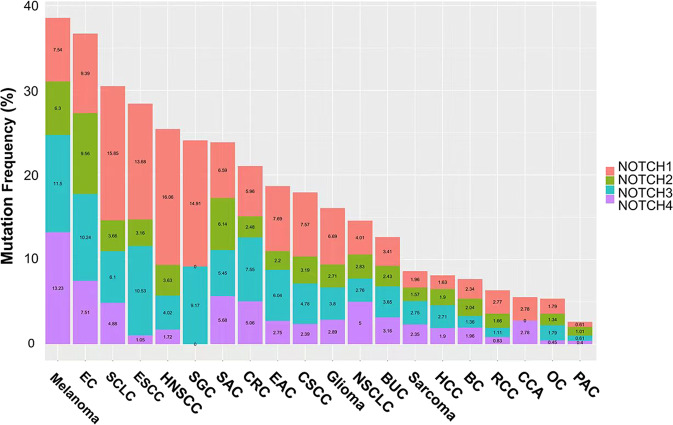
Mutation frequencies of NOTCH receptors in different cancers. Data are obtained from cBioPortal (http://cbioportal.org). We included data from two studies: MSK-IMPACT Clinical Sequencing and TCGA PanCancer Atlas Studies, with a total of 21289 patients. And we only used samples with mutation information, including missense, truncating, inframe, splice, and structural variation/fusion. This figure shows the mutation frequency of the four receptors of NOTCH in different cancer types. EC, endometrial carcinoma; SCLC, small-cell lung cancer; ESCC, esophageal squamous cell carcinoma; HNSCC, head, and neck squamous cell carcinoma; SGC, salivary gland cancer; SAC, stomach adenocarcinoma; CRC, colorectal cancer; EAC, esophagogastric adenocarcinoma; CSCC, cervical squamous cell carcinoma; NSCLC, non-small-cell lung cancer; BUC, bladder urothelial carcinoma; HCC, hepatocellular carcinoma; BC, breast cancer; RCC, renal cell carcinoma; CCA, cholangiocarcinoma; OC, ovarian cancer; PAC, prostate adenocarcinoma

NOTCH was first identified as an oncogene in T-ALL^342,343^. Subsequently, the alteration of NOTCH receptors was discovered in various cancers (Fig. 4). The activation of NOTCH in breast cancer, lung adenocarcinoma, hepatocellular cancer, ovarian cancer and colorectal cancer was determined to be oncogenic^78^ (Table 2). The pattern of NOTCH activation varies; for example, NOTCH can be activated by upstream signals or by structural alteration resulting from its internal mutations. Potential mechanisms of tumorigenesis include controlling the tumor-initiating cell phenotype, regulating known upstream or downstream tumor-associated signaling factors, such as MYC or P53, facilitating angiogenesis or tumor invasion, regulating the cell cycle, etc. These mechanisms will now be discussed based on cancer type.

#### Hematological malignancies

The oncogenic effects of *NOTCH* were first identified with the chromosome t (7;9) translocation of the *NOTCH1* gene in T-ALL^342,343^. More than 50% of T-ALL patients have *NOTCH1* somatic activating mutations^344^. Transplanted hematopoietic progenitor cells with constitutive activation of NOTCH1 signaling in murine models can lead to the development of T-ALL^345^. Mechanistically, NOTCH1 activation in T-ALL might involve the extracellular heterodimerization domain (HD) and/or the C-terminal PEST domain^344^. Mutations destabilizing the HD of *NOTCH1* could facilitate ligand-independent pathway activation. Furthermore, mutations disrupting the intracellular PEST domain could increase the half-life of NICD1. Many studies suggest that *NOTCH1* may induce the expression of *MYC* by regulating its enhancer N-Me and play a key role in the initiation and maintenance of T-ALL^346^. The interaction of *NOTCH1* and *PTEN* promotes anabolic pathways in T-ALL^347^. In addition to these synergistic effects, *NOTCH1* can directly regulate the expression of specific lncRNAs, such as LUNAR1, which is essential for the malignant proliferation of T-ALL cells^348^. Additionally, NOTCH signaling regulates the progression of the T-ALL cell cycle via the expression of the G(1) phase proteins cyclin D3, CDK4, and CDK6^349^. In recent years, activating mutations of *NOTCH3* independent of *NOTCH1* mutations have also been found in several cases^350^, providing novel insights into *NOTCH* mutations in T-ALL.

In addition, activating mutations in NOTCH have been identified in other hematological malignancies. Approximately 58% of splenic marginal zone lymphoma cases have activating NOTCH mutations, termed NNK-SMZLs, and such cases are related to inferior survival^351^. In a B cell chronic lymphocytic leukemia (B-CLL) murine model, dysfunction of NOTCH signaling reduces morbidity, while activation of NOTCH signaling increases the survival and apoptosis resistance of B-CLL cells^352^. In diffuse large B-cell lymphoma (DLBCL), *NOTCH* also participates in the tumor growth through the FBXW7-NOTCH-CCL2/CSF1 axis^353^. Although *NOTCH* plays an oncogenic role in most hematological malignancies, it inhibits the growth and survival of acute myeloid leukemia (AML), and consistent activation of *NOTCH1-4* leads to AML growth arrest and caspase-dependent apoptosis^354^.

#### Lung adenocarcinoma

In lung adenocarcinoma (LUAD) patients, high expression of NOTCH1 and NOTCH3 has been detected^355,356^. This alteration involves loss of NUMB expression, which increases NOTCH activity, and gain-of-function mutations of the *NOTCH1* gene^357^. In vivo and in vitro studies confirmed that *NOTCH1-3* contributes to the initiation and progression of LUAD^358–360^, indicating that NOTCH acts as an oncogene in LUAD. The tumorigenesis effect might involve activating mutations of downstream genes regulating the tumor-initiating cell phenotype. First, NOTCH3 is a key driver gene in *KRAS*-mediated LUAD that activates PKCι-ELF3-NOTCH3 signaling to regulate asymmetric cell division in tumor initiation and maintenance processes^361^. Second, coactivation of NOTCH1 and MYC increases the frequency of NICD1-induced adenoma formation and enables tumor progression and metastases in a mouse model^360^. In addition, NOTCH1 activation in *KRAS*-induced LUAD suppresses *p53*-mediated apoptosis^358^. However, NOTCH mutations have opposite effects in LUAD and squamous cell carcinoma (SCC) according to recent studies^362^. Since most studies of NOTCH are conducted in undistinguished non-small-cell lung cancer (NSCLC) patients, the specific effect of NOTCH in LUAD needs further research.

#### Colorectal cancer

Physiologically, NOTCH signaling is essential for the development and homeostasis of normal intestinal epithelia; for example, NOTCH signaling regulates the differentiation of colonic goblet cells and stem cells^363,364^. In human colorectal cancer (CRC) tissues, significant upregulation of NOTCH ligands (DLL1, DLL3, DLL4, JAG1, and JAG2) and aberrant activation of the NOTCH receptor (NOTCH1) are found^365,366^. Such abnormal NOTCH activation is associated with poorer prognosis and metastasis of CRC^367^. Inhibiting NOTCH by miR-34a and Numb suppresses the proliferation and differentiation of colon cancer stem cells^368^, indicating that NOTCH activation is a trigger of colon cancer development. Abnormal NOTCH signaling promotes the invasion and metastasis of CRC cells, possibly through the NOTCH-DAB1-ABL-TRIO pathway, EMT and TGF-β-dependent neutrophil effects^369^. On the one hand, NOTCH promotes CRC invasion by inducing ABL tyrosine kinase activation and phosphorylation of the RHOGEF protein TRIO^370^. On the other hand, active NOTCH signaling promotes the occurrence of metastasis by reshaping the tumor microenvironment and regulating EMT-associated transcription factors such as SLUG and SNAIL^367,371,372^. In conclusion, the NOTCH pathway induces EMT in colon cancer with *TP53* deletion^370,373,374^.

#### Breast cancer

Studies of NOTCH signaling in epithelial tumors were first performed in breast cancer^375–378^. Upregulation of non-mutated NOTCH signaling-related proteins, such as NOTCH1 and JAG1, is associated with poor prognosis in breast cancer^379^. In mouse models, mutations in *Notch1* and *Notch4* mediated by mouse mammary tumor viruses can promote epithelial mammary tumorigenesis^380,381^. Moreover, functionally recurrent rearrangements of *NOTCH* gene families are found in breast cancer, of which cell lines are sensitive to NOTCH inhibitors^382^. In HER2-expressing breast cancer cells, *NOTCH* activation seems to be associated with cytotoxic chemotherapy resistance^383^. Such an abnormal increase in NOTCH signaling expression is believed to be related to a lack of NUMB expression^384^, and its promoting effect on breast cancer tumorigenesis might be exerted from multiple aspects. First, NOTCH signaling maintains the stemness of breast cancer cells and promotes initiation^385,386^. Second, NOTCH signaling shapes elements of the breast cancer microenvironment, especially tumor-associated macrophages (TAMs), which is related to the innate immune phenotype^387^. In addition, NOTCH can be activated by the ASPH-Notch axis, providing materials for the synthesis/release of prometastatic exosomes in breast cancer^388^.

#### Ovarian cancer

In ovarian cancer, approximately 23% of patients have NOTCH signaling alterations^389^. *NOTCH1* and *NOTCH3* have been discovered to directly promote the occurrence and development of ovarian cancer^389–392^. Overexpression of NOTCH3 is related to cell hyperproliferation and apoptosis inhibition, as well as tumor metastasis and recurrence^393,394^. As NOTCH3 is positively correlated with JAG1 and JAG2 expression in ovarian cancer, the carcinogenic function of *NOTCH3* is potentially mediated by JAG1-NOTCH3 activation^395^, and dynamin-dependent endocytosis is required. Notch2/Notch3 and other NOTCH signaling molecules have achieved certain effects by inhibiting *Jag1* in a mouse ovarian cancer model^396,397^. In addition, through methylation of the VEGFR2 promoter, NOTCH signaling facilitates angiogenesis in ovarian cancer mediated by VEGFR2 negative feedback^398^.

#### Hepatocellular carcinoma

NOTCH signaling is a pathogenic factor in NASH, yet its role in hepatocellular carcinoma (HCC) is less well defined^399^. Approximately 30% of human HCC samples have activated NOTCH signaling, which in mice promotes the formation of liver tumors^400^. Recently, NOTCH activation was found in some HCC subtypes with unique molecular and clinicopathologic features and was found to be associated with poor prognosis^399^. *NOTCH* activation is also related to the activation of insulin-like growth factor 2, which contributes to hepatocarcinogenesis^401^. Furthermore, *NOTCH* activation facilitates EMT progression and metastasis in HCC^402^. On the other hand, NOTCH activation slows HCC growth and can predict HCC patient prognosis^403^. Mutations in the NOTCH target gene *HES5* in HCC samples can present both protumorigenic and antitumorigenic functions^404^. A close relationship between the function of *NOTCH1* and the *P53* mutation state has been reported, in which NOTCH1 activation increases the invasiveness of *P53* WT HCC cells while decreasing that of *P53*-mutated HCC cells^405^. Although showing contradictory functions in HCC, NOTCH is still mainly considered an oncogenic factor.

#### Glioma

NOTCH signaling used to be considered oncogenic in glioma, in which it maintains brain cancer stem cells^406^. Knockdown of NOTCH ligands in human brain microvascular endothelial cells (hBMECs) or inhibition of NOTCH signaling with a γ-secretase inhibitor in glioma constrains tumor growth both in vitro and in vivo^407,408^. *Notch1* has potentially oncogenic effects in the brain in association with other oncogenic hits, such as *p53* loss in a medulloblastoma mouse model^409^. Positive feedback of NOTCH1-SOX2 enhances glioma stem cell invasion along white matter tracts^410^. NOTCH also induces the expression of lncRNA and TUG1 to maintain the stemness of glioma stem cells and suppress differentiation^411^. Moreover, NOTCH1 signaling promotes the invasion and growth of glioma-initiating cells by modulating the CXCL12/CXCR4 chemokine system^412^. However, NOTCH suppresses forebrain tumor subtypes. Inactivation of Rbpj, Notch1, or Notch2 receptors accelerates tumor growth in a mouse model^413^. Such a subtype-specific effect of NOTCH in glioma might be related to cooperation with *P53*. Overall, NOTCH signaling acts either as an oncogenic factor or a tumor suppressor in different glioma subtypes, and the mechanisms need further exploration^414^.

#### Other cancers

Adenoid cystic carcinoma (ACC), commonly found in the salivary gland, frequently features activating *NOTCH1* and *NOTCH2* mutations^415–418^. NOTCH1 inhibitors have significant antitumor efficacy in both ACC patients and patient-derived xenograft (PDX) models^419,420^. Upregulation of *MYB* signaling through NOTCH mutation and amplification might also be a potential driving mechanism of ACC^421^. Activated NOTCH1 also produces CD133(+) ACC cells, regarded as cancer stem-like cells in ACC. In clear cell renal cell carcinoma (CCRCC), the overexpression of NOTCH ligands and receptors is observed in tumor tissues. Activated *NOTCH1* leads to dysplastic hyperproliferation of tubular epithelial cells, and treatment involving a γ-secretase inhibitor leads to CCRCC cell inhibition both in vitro and in vivo^422^.

### NOTCH as a tumor suppressor in cancers

NOTCH may be involved in many cancers as a protumor effector, but it can also act as a tumor suppressor in others, such as squamous cell carcinoma (SCC) and neuroendocrine tumors^423^ (Fig. 4, Table 2). Antitumor mechanisms include regulating transcription factors with malignant effects, activating downstream suppressive genes, inhibiting the cell cycle, etc. In light of studies regarding its antitumor effects, the traditional opinion of NOTCH as an oncogene has been challenged^414^.

#### Neuroendocrine tumors

NOTCH is now believed to act as a suppressor in neuroendocrine tumors (NETs), including tumors derived from the thyroid, neuroendocrine cells of the gut, the pancreas, and the respiratory system^424^. Small-cell lung cancer (SCLC) is the most common type of pulmonary NET, with nearly 25% of human SCLC cases presenting inactivation of NOTCH target genes in one comprehensive genomic profiling analysis^425^. A recent study used a multiomics approach to analyze the dynamic changes during transdifferentiation from NSCLC to SCLC^426^, which is a special feature of acquired resistance to EGFR-TKIs in LUAD. This study found that the downregulation of NOTCH signaling was essential for the initial cell state switch of LUAD cells^426^, indicating that NOTCH plays a tumor-suppressive role in SCLC. Furthermore, high DLL3 expression is frequently detected in SCLC and lung carcinoid tumors^55,426–428^, which downregulates NOTCH signaling via cis-inhibition. In an SCLC mouse model, activation of *Notch1* or *Notch2* reduces the expression of synaptophysin and Ascl1, inhibiting the cell cycle process^429,430^. Likewise, in human medullary thyroid cancer (MTC) tumor samples, NOTCH1 protein is undetectable, while the expression of NICD1 inhibits MTC cell proliferation^431^. In an analysis of gastroenteropancreatic NET tumor specimens, reduced NOTCH expression and mutated components were found^432,433^. Mechanistically, some studies consider that such an antitumorigenesis effect might be mediated by the NOTCH-ASCL1-RB-P53 tumor suppression pathway^434,435^, while others hold that activated NOTCH could inhibit cell growth via cell cycle arrest associated with upregulated P21^431,436^. NOTCH could also mark and initiate deprogramming in rare pulmonary NET cells that serve as stem cells in SCLC^437^. Considering the suppressor effect of NOTCH in NETs, drugs targeting DLL3 have been tested in SCLC, with promising results witnessed in preclinical trials (discussed in detail in the following sections).

#### Squamous cell cancers

In SCC specimens, inactivated *NOTCH1-3* has been detected^438–440^. 40% of head and neck squamous cell cancer (HNSCC) cases are found to have inactivated NOTCH1^441,442^. In cutaneous squamous cell cancer (cSCC) and its adjacent normal tissue, NOTCH receptors are also frequently found mutated, resulting in loss of function or downregulation^443^. Similarly, malfunction of NOTCH1 and NOTCH2 was found in lung squamous cell carcinoma (LUSC) patients^444^. This negative relation between NOTCH and carcinogenesis was also found in bladder^445^, esophageal^446,447^, and cervical SCC^448^. In an SCC mouse model, genomic aberrations in *NOTCH1* induced by mutagenic agents result in an increased tumor burden^449,450^. Dominant-negative Mastermind-like 1 (DNMAML1), an inhibitor of canonical NOTCH transcription, promotes de novo SCC formation^451^. Moreover, a study of γ-secretase inhibitors in Alzheimer’s disease (AD) patients showed that inhibiting S3 cleavage in NOTCH might increase the risk of nonmelanoma skin cancer^452^. Most studies of the mutated form of NOTCH in SCCs show that NOTCH function relies deeply on context; for example, NOTCH function can be affected by factors such as the P53 pathway and the intrinsic transcription-repressive protein RBP-Jκ^440^. The detailed regulatory mechanism is unclear, although some studies believe that NOTCH signaling maintains the CD133 phenotype in stem cells of SCCs^453^. Furthermore, decreased NOTCH1 expression also dysregulates cell cycle-associated genes in SCCs such as LUSC^362^.

#### Pancreatic ductal carcinoma

NOTCH mutation is common in PDAC^454^. *NOTCH1* can inhibit the formation of pancreatic intraepithelial neoplasia (PanIN) in a PDAC mouse model^455^. Additionally, *Notch1* loss is required progression in a *Kras*-induced PDAC mouse model^456^, suggesting its role as a tumor suppressor gene. However, previous studies suggest that NOTCH plays an oncogenic role in the occurrence and development of PDAC^457–459^. NOTCH signaling has been found to be activated in PDAC, which causes the growth of premalignant PDAC cells^457^.

**Table 2:** NOTCH Signaling in Cancers

Cancer type	Involved NOTCH components	Relevant evidence	Ref.
*NOTCH signaling pathway plays an oncogenic role*			
T-cell acute lymphoblastic leukemia	NOTCH1, NOTCH3	More than 50% of T-ALL patients have *NOTCH1* somatic activating mutations; Transplanted hematopoietic progenitor cells with activation of *Notch1* signaling in murine models can develop T-ALL; Activating mutations of *NOTCH3* without *NOTCH1* has also been found in several T-ALLs.	^344,345,350^
Splenic marginal zone lymphoma	NOTCH1, NOTCH2	Activating mutations of *NOTCH* signaling appeared in 58% of SMZLs, related to inferior survival.	^351^
B-chronic lymphocytic leukemia	NOTCH1-2, JAG1-2	Constitutively expression of NOTCH1, NOTCH2 proteins and their ligands JAG1 and JAG2 were detected in B-CLL; Dysfunction of NOTCH signaling reduces the morbidity of B-CLL, while activation of NOTCH signaling increases its survival.	^352,664^
Lung adenocarcinoma	NOTCH1, NOTCH3	*NOTCH1* and *NOTCH3* were detected highly expressed, suggesting poor prognosis and intensive invasion; *Notch1-3* were confirmed contributing to the initiation and progression of LUAD in vivo and in vitro*.*	^355,356,358^
Breast cancer	NOTCH1, NOTCH4, JAG1	Upregulation of non-mutated *NOTCH1* and *JAG1* is associated with poor prognosis of BC; The mutations of *Notch1* and *Notch4* mediated by the mouse mammary tumor virus can promote epithelial mammary tumorigenesis; BC cell lines with functionally recurrent rearrangements of *NOTCH* genes are sensitive to NOTCH inhibitors.	^379,380,382^
Colorectal cancer	NOTCH1	Upregulation of NOTCH ligands (DLL1, DLL3, DLL4, JAG1 and JAG2) and aberrant activation of NOTCH1 were detected; Active *Notch1* signaling induces the proliferation and activation of colon cancer hepatocytes, promoting cell invasion and metastasis.	^365,367^
Ovarian cancer	NOTCH1, NOTCH3	*Ntch1* and *Notch3* promote the occurrence and development of ovarian cancer; Overexpression of Notch3 is related to cell hyperproliferation and anti-apoptosis.	^389–393^
Adenoid cystic carcinoma	NOTCH1-2	Activated mutations of *NOTCH1* and *NOTCH2* were frequently detected in ACC; *NOTCH1* inhibitors have significant antitumor efficacy in both ACC patients and PDX models.	^415–420^
Clear cell renal cell carcinoma	NOTCH1	Overexpression of NOTCH ligands and receptors were observed in CCRCC tissues, and activated *NOTCH1* led to dysplastic hyperproliferation of tubular epithelial cells.	^422^
Hepatocellular carcinoma*	NOTCH1	Approximately 30% of human HCC samples have activated *NOTCH* signaling, promoting the formation of liver tumors in mice; *NOTCH* activation facilitates EMT progression and metastasis in HCC; Mutations in the NOTCH target gene *HES5* in HCC samples can present both protumorigenic and antitumorigenic functions.	^400,402,404^
Glioma*	NOTCH1-2	Inhibiting NOTCH signaling with a γ-secretase inhibitor in glioma constrains tumor growth both in vivo and in vitro. *NOTCH1* has oncogenic potential in the brain associating other oncogenic hotspots, such as p53 loss. Positive feedback of *NOTCH1-SOX2* enhances glioma stem cell invasion along white matter tracts. Inactivation of *Rbpj, Notch1 or Notch2* accelerates tumor growth in a mouse model.	^407–410^
*NOTCH signaling pathway palys a tumor suppressing role*			
Squamous cell cancers	NOTCH1-3	Inactivated *NOTCH1-3* were detected in SCC specimens; The genomic aberrations in *NOTCH1* induced by mutagenic agent could cause an increasing tumor burden in SCCs; DNMAML1, an inhibitor to canonical NOTCH transcription, promotes de novo SCC formation.	^438–440,449,451^
Neuroendocrine tumors	NOTCH1, DLL3	Nearly 25% of human SCLC cases present inactivation of NOTCH target genes; DLL3, an inhibitory NOTCH signaling components, was detected highly expressed in SCLC and lung carcinoid tumors; Gastroenteropancreatic and lung neuroendocrine tumors exhibit decreased NOTCH expression and mutated NOTCH components; Activating *NOTCH1* could inhibit the growth of thyroid neuroendocrine cancer cells in vitro.	^425,426,431,432^
Pancreatic ductal adenocarcinoma^a^	NOTCH1	*Notch1* could inhibit the formation of pancreatic intraepithelial neoplasia in a PDAC mouse model; *Notch1* loss is required for progression in a *Kras*-induced PDAC model.	^454–456^

*T-ALL* T-cell acute lymphoblastic leukemia, *SMZL* splenic marginal zone lymphoma, *B-CLL* B-cell chronic lymphocytic leukemia, *LUAD* lung adenocarcinoma, *BC* breast cancer, *ACC* adenoid cystic carcinoma, *PDX* patient-derived xenograft; *CCRCC* clear cell renal cell carcinoma, *HCC* hepatocellular carcinoma, *EMT* epithelial–mesenchymal transition, *SCC*, squamous cell cancer; *SCLC* small-cell lung cancer, *DANMAML1* Dominant-Negative Mastermind Like1, *PDAC* pancreatic ductal adenocarcinoma

^a^NOTCH might act as a tumor suppressor in oncogenic-oriented HCC^405^ and GBM^413^, while as an oncogene in tumorsuppressive-oriented PDAC^454–456^

### NOTCH signaling in the tumor microenvironment

**Fig. 5:**
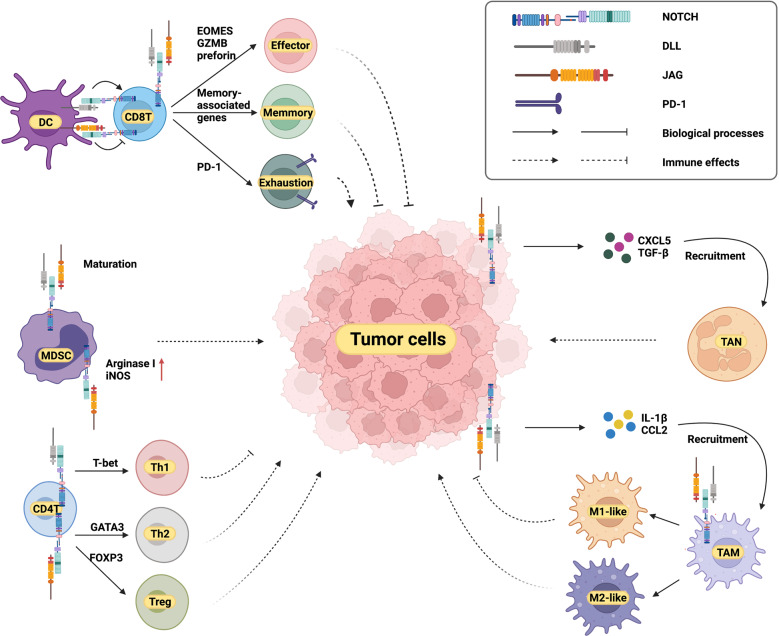
NOTCH signaling pathway in antitumor immunity. NOTCH signaling plays important roles in both tumor-suppressive and tumor-promoting immune cells. NOTCH signaling promotes the differentiation of many immune cells. DLL and JAG mediate both similar and distinct effects. DC, dendritic cell; CD8T, CD8+ T cell; MDSC, myeloid-derived suppressor cell; CD4T, CD4+ T cell; Th1, type1 T helper cell; Th2, type2 T helper cell; Treg, regulatory T cell; TAM, tumor-associated macrophage; TAN, tumor-associated neutrophil; PD-1, programmed death-1; EOMES, eomesodermin; GZMB, granzyme B; DLL, delta-like ligand; CCL2, C-C motif chemokine ligand 2

The tumor microenvironment (TME) refers to the factors surrounding tumor cells during their generation and development, including various immune cells, fibroblasts, extracellular matrix (ECM) components, and vasculature^460,461^. NOTCH signaling is deeply involved in regulating the diversified components of the TME^462^ (Fig. 5).

#### NOTCH signaling in immune cells

Generally, immune cells in the TME can be classified into two clusters, inflammatory (tumor-suppressive) immune cells and immune-suppressive (tumor-promoting) immune cells^463^, and NOTCH signaling plays important roles in both cell types. NOTCH signaling not only determines the differentiation of immune cells but also regulates their functional states.

##### Dendritic cells

In a mouse model with CD11c lineage-specific deletion of *Dll1*, CD8+ T cells are decreased, while regulatory T cells (Tregs) and myeloid-derived suppressor cells (MDSCs) are increased, leading to faster tumor growth^464^. Administration of a DLL1 analog can reverse *Dll1* deficiency-induced immunosuppression^464^. However, mice with CD11c lineage-specific deletion of *JAG2* do not show this phenotype, and administration of a JAG1-competitive antagonist reduces Tregs, improving antitumor immunity^464^. In the colitis-associated colorectal cancer (CRC) model, *Notch2* deficiency in the CD11c lineage impairs dendritic cell (DC) differentiation, reduces DC migration, and suppresses antigen-presenting capacity^465^, mirroring those conditions found in a pioneering study in nontumoral conditions^240^. In conclusion, both NOTCH ligands (DLL1) and receptors (NOTCH2) play positive roles in DC function, while JAG2 on DCs plays negative roles. As NOTCH signaling is crucial for DC differentiation and maturation, two research groups developed a method to increase the yield of cDC1s from mouse and human hematopoietic progenitor cells by employing DLL1-expressing stroma^466,467^, which might be applicable for autologous DC-based vaccination^468^.

##### CD8+ T cells

First, the DLL1-NOTCH1/2 axis is necessary for naïve CD8+ T cells to differentiate into effector T cells because it regulates the expression of the transcription factor eomesodermin (EOMES) and effector molecules (granzyme B and perforin)^469–472^. Selective activation of DLL1/4-NOTCH inhibits tumor growth^473^. In addition, NOTCH signaling is involved in the TCR-mediated self-amplification of T cells (section “The noncanonical NOTCH signaling pathway”). The activated TCR/CD3 complex can directly promote the cleavage of NOTCH receptors on endosomes, initiating the response of CD8+ T cells independent of NOTCH ligands^87^. As adenosine A2A receptor (A2AR) stimulation decreases TCR-mediated NOTCH activity^474^, inhibiting A2AR might help boost the CD8+ T cell response^41^. Second, NOTCH signaling is essential for the persistence and function of human lung tissue-resident memory T cells (TRM cells)^475^, thus assisting long tumor control^476–478^. Third, NOTCH signaling is also reported to have a negative impact on CD8+ T cells. NOTCH signaling upregulates the PD-1 expression of CD8+ T cells, thus promoting their exhaustion^479^. Inhibition of the NOTCH signaling pathway decreases the PD-1 level of CD8+ T cells and promotes the cytotoxicity of tumor-infiltrating CD8+ T cells in CRC patients^480^. Collectively, NOTCH receptors on CD8+ T cells play positive roles in antitumor immunity, paving the way for displaying NOTCH receptors on T cells for autologous T cell transfer therapy. One challenge in current chimeric antigen receptor-T (CAR-T) cell therapy is the exhaustion of transferred CAR-T cells. In light of this challenge, researchers designed new CAR-T cells with a synthetic NOTCH (synNOTCH) receptor loaded on the cell membrane^481,482^. These synNOTCH CAR-T cells not only promote the immune response but also maintain a higher fraction of effector T cells in the memory state^481,482^, which suggests the utility of such a strategy for next-generation CAR-T cell engineering^483,484^.

##### CD4+ T cells, B cells, and NK cells

Different ligand-mediated NOTCH signaling pathways also induce further differentiation and functions of CD4+ T cells^485^. DLL-mediated NOTCH signaling promotes type1 T helper cell (Th1) differentiation, while JAG1/2-mediated NOTCH signaling induces the differentiation of Th2 and Tregs^485–487^. Blocking NOTCH signaling with a GSI deeply impaired the generation and immunosuppressive function of Tregs^488^. However, Charbonnier et al. found that deletion of NOTCH components enhanced the immune-suppressive functions of Tregs, while transgenic overexpression of the NOTCH1 intracellular domain impaired Treg fitness^489^. As NOTCH signaling plays diversified roles in the generation and function of Tregs, distinguishing different signal-sending cells, ligands and receptors might be of much significance. DLL1-NOTCH2 signaling also mediates the development of splenic MZB cells. NK cells isolated from cancer patients show lower expression levels of NOTCH receptors than those of healthy donors^490^.

##### Tumor-associated macrophages

First, NOTCH signaling is necessary for the terminal differentiation of tumor-associated marcrophages (TAMs)^491^. The deletion of CSL in monocyte lineages abrogates TAM differentiation and functions^491^. A recent study found that inhibition of NOTCH signaling indeed impeded the differentiation of monocyte-derived TAMs while increasing the differentiation of Kupffer cell-like TAMs (kclTAMs) by upregulating Wnt/β-catenin signaling^492^. Second, NOTCH signaling participates in the recruitment of TAMs in basal-like breast cancer (BLBC)^387^. JAG1-NOTCH1/2/3 signaling in BLBC cells promotes the secretion of IL-1β and CCL2, recruiting TAMs into the TME. Simultaneously, the TAMs secrete transforming growth factor-β (TGF-β) to induce JAG1 expression in BLBC cells via the TGFβR1-SMAD2/3 pathway. This paracrine loop contributes to the suppressive immune microenvironment of BLBC and also indicates therapeutic opportunities. Third, NOTCH signaling regulates the polarization of TAMs between M1-like (tumor-suppressive) and M2-like (tumor-promoting) phenotypes. JAG1-NOTCH signaling between endocrine-resistant breast cancer cells and TAMs results in the differentiation of TAMs toward an M2-like phenotype, contributing to resistance to endocrine therapy^493^. NOTCH signaling mediates M2 polarization of TAMs in diffuse large B cell lymphoma (DLBCL) through the CREBBP/EP300-FBXW7-NOTCH-CCL2/CSF1 pathway^353^. However, NOTCH signaling is also reported to promote the M1 polarization of macrophages in anti-infection immunity^494,495^ and anticancer immunity^496,497^. In terms of transplanted tumors, macrophages with insufficient NOTCH signaling exhibit M2 phenotypes, while macrophages with forced activation of NOTCH signaling show M1 phenotypes and promote tumor shrinkage^496,497^.

##### Myeloid-derived suppressor cells

Similar to its role in TAMs, NOTCH signaling also participates in the differentiation^498–500^, chemotaxis^501^, and function of MDSCs. Regarding functional regulation, tumor-derived factors upregulate JAG1/2 on MDSCs through NFkB-p65 signaling, forming a suppressive immune microenvironment^502^. Anti-JAG1/2 antibodies decrease the accumulation and tolerogenic activity of MDSCs and inhibit the expression of the immunosuppressive factors arginase I and iNOS, thus restoring defective antitumor immunity^502^. In addition to its immune-regulatory functions, NOTCH signaling also participates in the MDSC-mediated regulation of tumor cell behaviors. Bone marrow-derived CD11b+JAG2+ cells infiltrate primary colorectal tumors and initiate the EMT program of tumor cells, thus promoting tumor metastasis^503^. Polymorphonuclear-MDSCs (PMN-MDSCs) interact with circulating tumor cells (CTCs) through NOTCH signaling, enhancing CTC dissemination and metastatic potency^504^. MDSCs activate NOTCH signaling in tumor cells to endow them with stem cell-like qualities in breast cancer^505,506^. In summary, NOTCH signaling mainly promotes the immune-suppressive and tumor-promoting functions of MDSCs; thus, targeting JAG1/2 might be a promising strategy.

##### Tumor-associated neutrophils

Jackstadt et al. reported that NOTCH1 signaling in CRC cells could promote the secretion of CXCL5 and TGF-β, recruiting tumor-associated neutrophils (TANs) to drive metastasis^367^. Additionally, JAG2-expressing TANs impair the cytotoxicity of CD8+ T cells via NOTCH signaling^507^.

#### NOTCH signaling in cancer-associated fibroblasts and the extracellular matrix

On the one hand, NOTCH signaling participates in the differentiation of cancer-associated fibroblasts (CAFs). In keratinocyte tumors, loss of NOTCH signaling promotes CAF differentiation and further tumor initiation^508–510^. However, in colon and prostate cancer, CAF differentiation is initiated by elevated NOTCH signaling^511,512^. In addition, CAFs activate NOTCH signaling in cancer cells to promote various malignant behaviors, including the cancer stem cell phenotype^513–515^, chemotherapy resistance^516^, metastasis^517,518^, and disease recurrence^519^. ECM components, such as fibulin-1^520^, fibulin-3^521^, microfibril-associated glycoprotein 2 (MAGP2)^522^, and laminin α5 (LAMA5)^523^, can also regulate the intensity of NOTCH signaling in cancer cells. Furthermore, activated NOTCH signaling in PDAC cells is reported to reshape the ECM through exosomes, thus promoting lung metastasis^524^.

#### NOTCH signaling in the tumor vasculature

The balance of DLL4 and JAG1 endothelial expression is important for tumor vasculature generation. When DLL4 is inhibited, small blood vessel branches sprout, tumor vascular density increases, vascular function remains poor, overall tumor perfusion decreases, and tumor growth is inhibited. Such effects on the tumor vasculature thus could be employed for antitumor therapy^525,526^. After binding to NOTCH receptors, JAG1 promotes angiogenesis by competing with DLL4. In breast cancer, JAG1 has been confirmed to induce tumor angiogenesis and tumor growth^527,528^. Additionally, NOTCH activation in ECs promotes lung metastasis, while endothelial NOTCH1 activation in the liver reduces intercellular adhesion molecule-1 expression and endothelial tumor cell adhesion and retention, thereby reducing liver metastasis^528,529^. During radiotherapy, endothelial NOTCH1 activation protects tumor vessels from radiotherapy-induced damage and regulates endothelial-mesenchymal transition^530^. Surprisingly, NOTCH3 acts as a receptor-dependent receptor in the endothelium to induce endothelial cell apoptosis and can be blocked by JAG1^526^. Furthermore, NOTCH blockade in VSMC-DA suppresses the contractile phenotype and promotes the secretory phenotype of VSMC-DA cells, thereby enhancing tumor cell invasion and proliferation^526^.

## Notch-targeted therapies

As a classical and fundamental signaling pathway in humans, NOTCH is crucial for the development and homeostasis of most tissues. Deregulated NOTCH signaling leads to various diseases, as presented above. For decades, NOTCH-targeting therapeutic strategies have been searched, with many drugs being studied in the preclinical stage or tested in clinical trials. NOTCH signaling has been investigated as a therapeutic target for the treatment of cancer, most recently in the fields of immunity and inflammatory disorders. In the following chapter, research on ongoing or completed NOTCH-targeted therapeutics will be presented according to the employed mechanism (Table 3).

**Table 3:** Drugs targeting the NOTCH signaling pathway assessed in clinical trials

Type	Drugs	NCT/Ref.	Year	Phase	Status	Cancer type and patients	Results
GSI	PF-03084014	NCT00878189^560^	2009	I	Completed	Solid malignancies, *N* = 64	ORR: 13%; 1 CR observed in patients with advanced thyroid cancer, and 5 PRs in patients with desmoid tumors; All-grade AEs: 84.4%, grade ≥ 3 AEs: 35.9%.
		NCT00878189^665^	2009	I	Completed	T-ALL and T-LBL, *N* = 8	1 CR in a T-ALL patient with NOTCH1 mutation.
		NCT02299635	2015	II	Terminated	TNBC, *N* = 19	SAEs: 6/19; study terminated prematurely based on project reprioritization by the sponsor.
		NCT01981551^571^	2013	II	Active	Desmoid tumors (aggressive fibromatosis), *N* = 17	5 (29%) patients experienced a PR for more than 2 years with tolerable toxicity.
		NCT04195399	2020	II	Recruiting	Progressive, surgically unresectable desmoid tumors, *N* = 35	-
	RO4929097	NCT00532090^561^	2007	I	Completed	Platinum-resistant ovarian cancer, *N* = 110	1 PR in patients with colorectal adenocarcinoma with neuroendocrine features; 1 nearly complete FDG-PET response in a patient with melanoma.
		NCT01119599^562^	2010	0/I	Completed	Glioma, *N* = 21	No dose-limiting toxicities were observed in combination with temozolomide; decreased expression of NICD in tumor cells and blood vessels.
		NCT01175343^569^	2010	II	Completed	Platinum-resistant ovarian cancer, *N* = 45	No objective responses were observed.
		NCT01122901^666^	2010	II	Completed	GBM, *N* = 47	Inactive in recurrent GBM patients.
		NCT01120275^568^	2016	II	Completed	Metastatic melanoma, *n* = 32	Tolerated but did not achieve NOTCH target inhibition.
		NCT01116687^570^	2010	II	Completed	Metastatic colorectal cancer, *N* = 37	No radiographic responses were seen, and time to progression was short.
	MK-0752	NCT00100152	2005	I	Terminated	T-ALL, *N* = 50	1/6 patients showed 45% reduction in mediastinal mass; study was halted for severe diarrhea.
		NCT00106145^667^	2005	I	Completed	Solid tumors, *N* = 103	1 objective response and 10 cases of SD were observed in patients with high-grade gliomas; weekly dosing was generally well tolerated.
		NCT00572182^566^	2008	I	Terminated	Brain and central nervous system tumors, *N* = 33	No objective responses were reported in 23 pediatric patients; study terminated by sponsor.
		NCT00645333^668^	2008	I/II	Completed	Breast cancer, *N* = 30	Enhanced the efficacy of docetaxel with manageable toxicity.
		NCT00756717	2008	IV	Completed	Breast cancer, *N* = 20	No serious adverse events; No available efficacy data..
	LY3039478	NCT01695005^565,669,670^	2012	I	Completed	Solid cancers, *N* = 237	Prednisone might reduce gastrointestinal toxicities; PR was observed in 1 patient with breast cancer, 1 patient with leiomyosarcoma and 1 patient with angiosarcoma.
		NCT02518113^671^	2015	I	Completed	T- ALL/T-LBL, *N* = 36	6 patients (16.7%) experienced DLTs; 1 patient (2.8%) had a confirmed response that lasted 10.51 months.
		NCT02784795^672^	2016	Ib	Completed	Solid cancer, *N* = 94	Combination with other anticancer agents produced disappointing results.
	LY900009	NCT01158404^564^	2010	I	Completed	Solid cancer, *N* = 35	No objective response; 5/35 patients had a SD.
	AL101	NCT04461600	2020	II	recruiting	NOTCH-activated TNBC, *N* = 67	-
		NCT04973683	2021	I	recruiting	NOTCH-activated ACC, *N* = 12	-
DLL3	Rovalpituzumab tesirine (Rova-T)	NCT01901653^673^	2013	I	Completed	SCLC, *N* = 74	11 (18%) patients had an objective response, ten of whom had high DLL3 expression; 28 (38%) suffered serious drug-related adverse events.
		NCT02819999^579^	2016	I	Terminated	SCLC, *N* = 26	There was no clear efficacy benefit of combining Rova-T with platinum-based chemotherapy.
		NCT03026166^589^	2017	I/II	Terminated	SCLC, *N* = 42	ORR was 30% in patients treated with combination therapy with Rova-T and ICIs; however, the toxicity was high, suggesting that the combination was not well tolerated; enrollment was stopped because of the DLT.
		NCT02674568^586^	2016	II	Completed	SCLC, *N* = 339	Median OS was 5.6 months; grade 3-5 AEs were seen in 213 (63%) patients; Demonstrated modest clinical activity in 3L+ SCLC, with associated toxicities.
		NCT03033511^587^	2017	III	Terminated	SCLC, *N* = 748	Lack of survival benefit of maintenance therapy with rovalpituzumab tesirine after first-line platinum-based chemotherapy; the study did not meet its primary end point and was terminated early.
		NCT03061812^588^	2017	III	Completed	SCLC, *N* = 444	Compared with topotecan, Rova-T exhibited an inferior OS and higher rates of serosal effusions, photosensitivity reactions, and peripheral edema.
	SC-002	NCT02500914^591^	2015	I	Terminated	SCLC, *N* = 35	5 (14%) patients achieved a PR; 37% of patients had serious AEs considered to be related to SC-002; no further development is planned because of the systemic toxicity and limited efficacy.
	AM757	NCT03319940	2017	I	Recruiting	SCLC, *N* = 332	-
	HPN328	NCT04471727	2020	I	Recruiting	SCLC, *N* = 67	-
DLL4	Enoticumab (REGN421)	NCT00187159^594^	2015	I	Completed	Solid tumors, *N* = 53	2 PRs were observed in patients with NSCLC and ovarian cancer; MTD was not reached.
	Demcizumab (OMP-21M18)	NCT00744563^595^	2014	I	Completed	Solid tumors, *N* = 55	Demonstrated antitumor activity with a low dose.
		NCT01189968^674^	2010	I	Completed	Metastatic nonsquamous NSCLC, *N* = 40	Modulated the expression of genes regulating NOTCH signaling and angiogenesis; increased the risk of cardiovascular disease when combined with pemetrexed and carboplatin.
		NCT01952249^596^	2013	Ib/II	Phase Ib, completed; phase II, terminated	Platinum-resistant ovarian, primary peritoneal, and fallopian tube cancer, *N* = 19	Researchers are no longer pursuing ovarian cancer as an indication; the phase II portion of the study was terminated.
NOTCH1	Brontictuzumab (OMP-52M51)	NCT01778439^420^	2013	I	Completed	Selected refractory solid tumors, *N* = 48	2 patients achieved PR and 4 patients achieved ≥ 6 months of SD in ACC with NOTCH1 activation; DLTs included diarrhea and fatigue.
NOTCH2/3	Tarextumab (OMP-59R5)	NCT01277146^616^	2011	I	Completed	Solid tumors, *N* = 42	9 subjects had SD; Lower doses were tolerated.
		NCT01647828^615^	2012	II	Completed	Untreated metastatic pancreatic cancer, *N* = 177	There were no OS, PFS, or ORR benefits with the addition of tarextumab to nab-paclitaxel and gemcitabine in first-line metastatic PDAC.
		NCT01859741	2019	I/II	Terminated	SCLC, *N* = 172	Terminated for unimproved PFS in combination with etoposide and platinum therapy.
NOTCH3	PF-06650808	NCT02129205^617^	2014	I	Terminated	Breast cancer and other advanced solid tumors, *N* = 40	5 PRs were observed with manageable safety; all of responders had positive NOTCH3 expression; the study was terminated due to a change in sponsor prioritization.

*T-ALL* T cell acute lymphoblastic leukemia, *T-LBL* T cell lymphoblastic lymphoma, *TNBC* triple-negative breast cancer, *SCLC* small-cell lung cancer, *NSCLC* non-small-cell lung cancer, *PDAC* pancreatic ductal adenocarcinoma, *GBM* glioblastoma, *ORR* objective response rate, *CR* complete response, *PR* partial response, *SD* stable disease, *PFS* progression-free survival, *OS* overall survival, *AE* adverse event, *SAE* serious adverse event, *ACC* adenoid cystic carcinoma, *FDG-PET* [18F]-2-fluoro-2-deoxy-D-glucose-positron emission tomography, *DLT* dose-limiting toxicity, *NICD* NOTCH intracellular domain, *3L+* more than 3 lines of therapy, *MTD* maximum tolerated dose

### Cleavage inhibitors

#### S1 cleavage

Precursors of NOTCH receptors require S1 cleavage in the Golgi before integration with their ligands. Sarcoendoplasmic reticulum Ca2+-ATPase (SERCA) is an important accessory factor in this process that modulates ATP-dependent calcium pumps^531^. Malfunction of SERCAs impairs NOTCH signaling, especially that of mutant *NOTCH1*^532^. Mutant NOTCH1 protein acts as an oncogene in T-ALL as well as other malignant tumors^533^, making SERCAs potential therapeutic targets^534^. Thapsigargin, a guaianolide compound of plant origin that inhibits SERCAs in mammalian cells, has been tested in breast cancer and leukemia at the preclinical stage^535–537^. CPA^534^, CAD204520^538^ and other small molecular inhibitors of SERCA with lower off-target toxicity have been investigated in the laboratory, yet no surprising results have been reported to encourage further clinical trials.

#### S2 cleavage

S2 cleavage occurs in the ligand–receptor binding domain, mediating ectodomain shedding and regulating the transmission speed of NOTCH signaling^539,540^. A disintegrin and metalloproteinase domain-containing protein 10 (ADAM10) or ADAM17 (also called tumor necrosis factor-alpha convertase, TACE) can be exploited to prevent S2 cleavage and NOTCH signaling transmission, as they are key enzymes of S2 cleavage^62,541–543^. Similar to SERCA inhibitors, ADAM inhibitors target the entire NOTCH pathway. Small molecule drugs targeting ADAMs have been studied in non-small-cell lung cancer^544^, hepatocellular carcinoma^545^, renal carcinoma^546^, breast cancer^547^, and systemic sclerosis^548^. Some of these inhibitors have shown anti-NOTCH activities in vitro and in animal experiments, yet no clinical trial has been initiated.

#### S3 cleavage

The canonical signal transmission of NOTCH signaling from outisde the cell to inside the cell relies heavily on S3 cleavage mediated by the γ-secretase complex^549,550^, suggesting that it is promising to modulate the function of γ-secretase for treatment.

##### γ-Secretase inhibitors

γ-Secretase inhibitors (GSIs) were first tested as a treatment for Alzheimer’s disease (AD) in clinical trials because γ-secretase contributes to catalyzing the production of β-amyloid peptide. Unfortunately, the study was terminated shortly after it began because of serious NOTCH-associated adverse events such as gastrointestinal symptoms, infections, and nonmelanoma skin cancers^551^. Since then, researchers have attempted to treat cancer with GSIs to disrupt NOTCH signaling. In preclinical studies, GSIs are widely studied as a treatment for cancer, showing antitumor activity in diverse tumor types, such as breast cancer^552,553^, hepatocellular carcinoma^554,555^, non-small-cell lung cancer^556^, colorectal cancer^557^, prostate cancer^558^, and gliomas^559^. Cancer patients were first documented to receive GSI treatment in 2006, with one of six patients with T-ALL or acute myeloid leukemia receiving MK-0752 in a phase I clinical trial; the trial showed a promising 45% reduction in mediastinal mass after 28 days, although the treatment was paused because of severe diarrhea (NCT00100152). Other drugs, including PF-03084014^560^, RO4929097^561,562^, BMS-986115^563^, LY900009^564^, LY3039478^565^, and MK-0752^566,567^, have emerged in phase I trials, all of which have shown antitumor efficacy. However, most have presented dose-limiting toxicities. To date, only RO4929097 and PF-03084014 have entered phase II trials. Unfortunately, although the adverse events (AEs) were well tolerated, only 1 patient among 32 patients with metastatic melanoma treated with RO4929097 achieved a partial response^568^. Similar outcomes occurred in platinum-resistant epithelial ovarian cancer and colorectal cancer, with no objective response among valid participants^569,570^; thus, few agents have entered phase III/IV clinical trials. PF-03084014, also called nirogacestat, achieved more promising outcomes in patients with desmoid tumors (aggressive fibromatosis) than RO4929097, as 29% of the 15 patients experienced a confirmed partial response that was maintained for more than 2 years^571^. A phase III clinical trial for nirogacestat has already been registered, although the trial has yet to begin (NCT03785964).

In addition to cancer, because NOTCH plays a critical role in the differentiation of Th cells, GSIs have also been studied in allergic diseases such as asthma^572^. NOTCH signaling regulates Th1 and Th2 responses in allergic pulmonary inflammation, indicating its promising targetability in immune disease.

##### γ-Secretase modulators

γ-Secretase modulators (GSMs) were originally studied in AD^573^. As a superior option to GSIs, GSMs aim to modify the catalytic activity of γ-secretase rather than to nonselectively inhibit it, enabling partial NOTCH signaling function to be maintained and thus theoretically ameliorating adverse events^574^. The selective inhibitor MRK-560 targeting PSEN1, an important catalytic subclass of γ-secretase complexes, has been proven to effectively decrease mutant *NOTCH1* processing and cause cell cycle arrest in T-ALL without associated gut toxicity^575^. GSMs are only applied in AD as drugs that are designed to modulate amyloid-β (Aβ) peptide generation without impacting the function of NOTCH^576,577^.

### Antibody-drug conjugates

Given the severe adverse events of inhibiting the overall NOTCH pathway, antibodies targeting different receptors and ligands have been explored to achieve precise targeting of NOTCH signaling^578,579^. There are five ligands and four receptors in the NOTCH signaling pathway^21^. Although the roles of each component are not completely clear, functions related to specific diseases have been confirmed, making them potential targets^41^.

#### Antibodies against ligands

##### JAG1

As reported previously, the upregulated expression of JAG1 enhances proliferation and angiogenesis in various malignant tumors, including adrenocortical carcinoma^580^, breast cancer^379^, and prostate cancer^581^. These pathological mechanisms make JAG1 a promising target, and monoclonal antibodies against JAG1 have been studied in breast cancer^582^, ovarian cancer^396^, and other malignant tumors^582^. 15D11, one of the most promising fully human monoclonal antibodies against JAG1, has been studied at the preclinical stage; 15D11 increases chemotherapy sensitivity, reduces neoplastic growth in bone metastases, and, most importantly, causes minor adverse effects^583^.

##### DLL3

DLL3 is an inhibitory ligand of NOTCH signaling that is highly upregulated and aberrantly expressed on the cell surface of small-cell lung cancer (SCLC) and other high-grade neuroendocrine tumors as a key driving gene^55,584,585^. DLL3-directed antibody-drug conjugates (ADCs) induce durable and safe responses in SCLC and large-cell neuroendocrine cancer (LCNEC) PDX tumor models^427^. Positive results inspired further clinical trials. In 2017, Charles M Rudin et al. first reported their encouraging results of rovalpituzumab tesirine (Rova-T); 11 of 60 assessable patients with SCLC or LCNEC had confirmed objective responses, and the objective response rate (ORR) was relatively higher in patients with high DLL3 expression. Although 38% of 74 patients suffered severe drug-related AEs, the AEs could be controlled^579^. Unfortunately, further phase II and III studies failed to achieve their efficacy end points. Relapsed/refractory SCLC patients receiving Rova-T after at least two lines of therapy achieved a median overall survival (mOS) time of only 5.6 months, and the ORR was 12.4%^586^. A study of Rova-T as a maintenance therapy after first-line platinum-based chemotherapy was terminated shortly after it began due to a lack of survival benefit^587^. Compared with concurrent standard second-line chemotherapy, Rova-T showed shorter OS and lower safety^588^. Attempts to combine chemotherapy and immune checkpoint inhibitors also failed, with extra toxicities and moderate efficacy^579,589^. Although the abovementioned studies failed to meet their expected end points, complete responses appeared in nearly every study, indicating that this therapeutic strategy has good prospects. However, strategies to stratify patients and appropriate biomarkers should be explored. Researchers have also attempted to explore further indications and novel drugs related to DLL3-targeting antibodies. IDH-mutant gliomas show selective and homogeneous expression of DLL3, and researchers found that patient-derived IDH-mutant glioma tumorspheres were sensitive to Rova-T in vitro^590^. Another DLL3 ADC, SC-002, presented an ORR of 14% and a severe AE rate of 37% in a phase I clinical trial in SCLC^591^. Furthermore, some novel drugs targeting DLL3 are in trials actively recruiting patients, such as AM757 (a bispecific antibody targeting DLL3 and CD3, NCT04702737) and HPN328 (a trispecific antibody, NCT04471727).

##### DLL4

DLL4 is an important regulator of tumor angiogenesis and cancer stem cells and is activated in a wide range of human cancers^592^. The combination of specific DLL4 blockade and ionizing radiation impairs tumor growth by promoting nonfunctional tumor angiogenesis and extensive tumor necrosis^593^. When combined with VEGF blockade, REGN421, a monoclonal antibody targeting DLL4, presented antitumor effects in ovarian cancer^525^. A phase I clinical trial of REGN421, also called enoticumab, was conducted in patients with advanced solid tumors. Of the 32 treated patients in whom toxicity was tolerable, 2 patients had partial response, and 16 patients had stable disease^594^. Demcizumab, another anti-DLL4 antibody, showed antitumor activity at the minimum dose and with shorter exposure in a phase I clinical study of solid tumors but presented a significant risk of cardiac toxicity^595^. After dose optimization, combining demcizumab with paclitaxel achieved an ORR of 21% in platinum-resistant ovarian cancer patients without dose-limiting toxicity^596^. Strategies employing dual variable domain immunoglobulin (DVD-Ig) molecules targeting DLL4 and VEGF have been studied, such as ABT-165, which showed superior efficacy and safety in preclinical models^597^, and navicixizumab (OMP-305B83), which presented modest antitumor potency and toxicity in a phase Ib clinical trial of solid tumors^598^.

##### JAG2/DLL1

JAG2, believed to promote cell survival and proliferation, interacts with NOTCH2, the nucleus pulposus (NP)^599^, and hematopoietic stem and progenitor cells (HSPCs)^600^. Additionally, high expression of JAG2 facilitates the development of cancers, such as lung adenocarcinoma^601^ and bladder cancer^602^. DLL1 is essential for the development and differentiation of B lymphocytes^227,603^. These two ligands might be promising targets, although drugs targeting these ligands have yet to be reported.

#### Antibodies against receptors

##### NOTCH1

Mutant *NOTCH1* induces the occurrence of T-ALL and T-ALL cell proliferation^344,604^. It can also act as an oncogene in colorectal carcinoma^605^, glioma^606^ and other malignant tumors^607^, making it a possible antitumor target. In phase I clinical trials, a monoclonal antibody targeting NOTCH1 called brontictuzumab was tested in patients with solid tumors (NCT03031691 and NCT01778439) and lymphoid malignancies (NCT01703572). A clinical benefit was achieved in 6 of 12 ACC patients with tolerable toxicity^420^. In addition to tumor activation, *NOTCH1* also promotes the immune response depending on Tregs. In preclinical trials, drugs selectively inhibiting NOTCH1 have been shown to strengthen the function of Tregs to suppress the progression of inflammatory arthritis^608^ and modulate the immune response in transplantation^609^.

##### NOTCH2/NOTCH3

Dysregulated NOTCH2 is vital for the development of cancers such as some B cell leukemias^610^, pancreatic ductal adenocarcinoma (PDAC)^611^, and malignant melanoma^612^. Similarly, NOTCH3 acts as a facilitating factor in various tumors, such as lung cancer^613^, ERBB2-negative breast cancer^614^, and ovarian cancer^391^. OMP-59R5 (tarextumab), which blocks both NOTCH2 and NOTCH3, is effective in treating a variety of tumors^397^ and has been tested as a treatment for PDAC^615^, SCLC (NCT01859741), and other solid tumors^616^ in clinical trials. However, OMP-59R5 in combination with chemotherapy did not produce a superior outcome in PDAC or SCLC patients, and neither drug achieved a better objective response in other solid tumors. PF-06650808, a novel anti-NOTCH3 ADC, achieved 5 partial responses among 40 patients with breast cancer or other solid tumors, with a manageable safety profile and positive NOTCH3 expression detected in all responders^617^.

##### NOTCH4

The functions of NOTCH4 differ in different types of cancer. The overexpression of NOTCH4 is regarded as a poor prognosis marker in some scenarios^618^, while in others, it is considered a favorable marker^619^. There are no mature drugs targeting NOTCH4.

### Transcription blockers

Activating the transcription of target genes is the last step of NOTCH signaling. Therapies targeting downstream mediators of NOTCH signaling remain unexplored. NOTCH transcription depends on the NOTCH ternary complex (NTC), which contains the DNA-binding protein CSL (also called CBF-1/RBPJ, Su (H), or Lag-1), NICD and MAML1^620,621^. RIN1, a small molecule inhibitor of RBPJ, causes proliferation of hematologic cancer cell lines in vitro^622^. IMR-1, a small molecule inhibitor of MAML1, inhibits the growth of NOTCH-dependent cell lines in vitro^623^. CB-103, an orally active small molecule altering NTC function, produces loss-of-function NOTCH phenotypes and inhibits the growth of human breast cancer and leukemia xenografts, notably without causing the dose-limiting intestinal toxicity of other NOTCH inhibitors^624^. Such novel drugs may represent new agents for NOTCH-based diseases.

### NOTCH signaling agonists

NOTCH signaling can both accelerate and suppress the development of diseases, which unsurprisingly applies in cancers^625,626^. That is, enhancing NOTCH signaling can be a targeted therapy strategy. Some chrysin and hesperetin compounds have been used to activate NOTCH signaling in anaplastic thyroid cancer with *NOTCH1* deficiency^627,628^. Inhibitory effects on established tumor cell lines were found, although the underlying mechanism remains unclear. The negative regulatory region (NRR) can autoinhibit the metalloprotease cleavage of NOTCH to enhance its signaling. Some activating antibodies of NOTCH receptors induce conformational changes in the NRR, making it accessible to ADAM metalloproteinases, thus facilitating activation of NOTCH signaling^629^.

### Summary of clinical trials

Several NOTCH-targeted therapies have been evaluated in clinical trials; specifically, these therapies have been tested in cancers^41^. Among cleavage inhibitors, drugs targeting S1-S2 cleavage are still within preclinical stages. Drugs targeting S3 cleavage (GSIs and GSMs) have made their way into further clinical research; research of GSIs has been restrained due to severe toxicities, though GSMs are being continuously explored. Among the antibodies against ligands, drugs targeting JAG1, DLL3 and DLL4 have shown promising results in preclinical studies. Drugs targeting DLL3 and DLL4 have been studied in early clinical trials, with only those targeting DLL3 performing well. Unfortunately, further studies of agents targeting DLL3 failed to meet expectations. Drugs targeting JAG2/DLL1 have shown great potential, but no drug has reached mature development. Among the antibodies against receptors, the majority have achieved mediocre results. Of the transcription blockers and signal agonists, the blockers have only been studied in the preclinical stage, while agonists remain only theoretical. Of the abovementioned agents, those targeting DLL3 and GSIs are the most popular because they have shown potential.

However, neither of these agents can be applied clinically considering safety and efficacy. On the one hand, most pan-NOTCH inhibitors exhibit dose-limiting gastrointestinal toxicities mediated by hyperplasia of intestinal goblet cells, including diarrhea and vomiting, which often lead to suspension of further investigations^253,630^. Regarding GSIs, attempts have been made to improve tolerance, such as combining GSIs with glucocorticoids^631^, using intermittent dosing regimens^632^, and applying drugs that inhibit disease-specific subunits of the γ-secretase complex^633^. On the other hand, the majority of ADCs have failed to reach the expected efficacy in cancer studies, although they have performed well in some individuals. Cell heterogeneity might be an explanation for such findings. Taking SCLC as an example, researchers found that a minority of nonneuroendocrine SCLC cells with NOTCH activation could sustain the growth of neuroendocrine SCLC cells without NOTCH activation and exhibit cancer stem cell-like properties^634^, resulting in primary resistance to anti-DLL3 drugs. Insufficient affinity of ADCs might be another reasonable explanation. Additionally, the complexity of NOTCH signaling and bypass signaling might circumvent NOTCH-targeted therapies. In the future, exploring predictive biomarkers, reducing drug toxicities, and exploiting multitargeted drugs might overcome the challenges of NOTCH-targeted therapies.

## Concluding remarks and future perspectives

It has been approximately 110 years since the NOTCH gene was first identified in *D. melanogaster*. We summarized both classical and cutting-edge findings of NOTCH signaling in this review, illustrating the history, architecture, regulatory mechanism, physiology, and pathology of NOTCH signaling as well as therapeutics targeting NOTCH signaling. We identified certain areas of basic research and clinical applications of NOTCH signaling as worthy of further exploration.

One of the most interesting things regarding NOTCH signaling is the dual role it plays in different conditions, particularly in cancers. First, the functions of NOTCH signaling are different within the same tissues, and this is possibly caused by the utilization of different ligands; for example, DLL4/JAG1 regulates tumor vasculature, and DLL1/JAG2 regulate DC functions. Second, the functions of NOTCH signaling vary in different tissues. For instance, NOTCH acts as an oncogene in some tumors and as a tumor suppressor gene in others. Several mechanisms might explain this phenomenon: (a) Different tissues have different expression patterns of NOTCH signaling components, and thus, the outcomes of NOTCH signaling are tissue-specific; for example, DLL3 has tissue-specific effects in SCLC, and NOTCH1 has tissue-specific effects in T-ALL. (b) NOTCH signaling effects occur over a small range, while the cell morphology and intercellular distance are diverse in different tissues. (c) NOTCH signaling activates the transcription of a series of genes containing both positive and negative regulators of biological events. As these downstream genes are also regulated by other driver genes, such as *Myc* and *P53*, the mutational status of these driver genes also affects the outcome of NOTCH signaling. Third, tumors are massive complexes containing different clones of cancer cells and multiple types of noncancerous cells, making the overall effect of NOTCH signaling complicated and unpredictable.

Several strategies can be employed to clarify the mechanisms of NOTCH signaling. First, deciphering the subtle differences between different ligand–receptor interactions is essential. Second, spatially resolved transcriptomic analyses^635^, which dissect the embedded tissues into very small pieces and acquire their expression profiles, can be used to explore the impact of spatial characteristics on the outcome of NOTCH signaling. Third, comprehensive analysis of NOTCH target genes is needed because there may be more target genes than are currently known^81^, and epigenetic and transcriptomic analyses might help.

NOTCH-targeted therapy has been studied for decades but has failed to meet expectations. The reasons for these shortcomings might be the cytotoxicity induced by pan-NOTCH inhibitors, the low affinity of current ADCs, and the upregulation of bypass pathways. Novel drugs such as isoform-specific drugs and high-affinity ADCs may be a solution, as they might have increased efficacy and lower cytotoxicity. In addition, protein refolding is an attractive mode of action to employ to restore the functions of inactivated NOTCH signaling. Another strategy is to develop novel treatment strategies, such as DC-pulsed vaccine therapy and synNOTCH CAR-T cell therapy. Complementary combination therapies, such as combination of inhibitors of other pathways, chemotherapy, radiation therapy, and immunotherapy, are also promising. Among these potential combinations, combinations with immunotherapy are expected to be the most useful.

Much work remains to be accomplished for combining NOTCH-targeted therapy with immunotherapy, and the following strategies might help. First, functional studies are needed to comprehensively delineate the consequences of different NOTCH mutations and their effects on the immune microenvironment. NOTCH plays a complex role in tumor immunity, and its overall impact on tumors remains unclear. Second, clinical applications targeting different stages and types of cancer should be considered separately. Canonical NOTCH signaling is widely activated among cells to mediate adjacent intercellular interactions, yet its effects are highly dependent on context and/or cancer type. Third, appropriate ligands and/or receptors should be well chosen because they may have contradictory biological effects. For example, DLL1-NOTCH mainly functions as an immune-activating signal in DCs and CD8+ T cells. However, JAG1/2-NOTCH mainly functions as an immunosuppressive signal, inhibiting DCs and CD8+ T cells while activating many immunosuppressive cells. It is evident that drugs selectively enhancing DLL1-NOTCH signaling while inhibiting JAG1/2-NOTCH signaling can outperform pan-NOTCH-targeting drugs in actual practice. Fourth, conditions triggering the anti-immune or proimmune effects of NOTCH signaling in tumor cells should be considered. It has been acknowledged that NOTCH signaling may be immunosuppressive or tumor suppressive, yet the conditions or triggering factors leading to certain effects remain unknown. Thus, the effect of NOTCH signaling under different microenvironments should be investigated to generate better and more predictable medical applications. Fifth, cytotoxicity should be considered, including the toxicity of the drug itself and the toxicities induced by combination therapies. Sixth, predictive biomarkers should be explored to bolster NOTCH-targeting monotherapy and/or ICI therapy should be combined with NOTCH-targeting monotherapy to achieve maximum efficacy.

In summary, NOTCH factors present complicated and highly changeable functions, suggesting that elaboration of the general mechanism is required. Novel drugs with higher efficacy and lower cytotoxicity are worth investigating, as are new therapeutic strategies. Once a complete understanding of NOTCH signaling is achieved, it can be applied in actual medical practice, fulfilling the long-overdue mission of benefiting patients.

## Supplementary information

Supplementary Table1. The role of NOTCH signaling in developmental processes
