# Supplementary material for: Notch signaling pathway: architecture, disease, and therapeutics
Source: Signal Transduct Target Ther. 2022 Mar 24;7:95. doi: 10.1038/s41392-022-00934-y (PMC8948217; doi:10.1038/s41392-022-00934-y)
Supplement: Supplementary file 1 — Supplementary Table1. The role of NOTCH signaling in developmental processes [file 41392_2022_934_MOESM1_ESM.docx]

**Supplementary Table1. The role of NOTCH signaling in developmental processes.**

| **Organ/Tissue** | **The role of NOTCH signal in development and repair** | **PMID** |
| --- | --- | --- |
| Somitogenesis | Determine the period of the split clock to form a delay-negative feedback loop;  Synchronizes the oscillations between cells. | 7789282  28942924  31915376 |
| Skeleton | Maintain the number and stemness of MPC;  Inhibit bone cell production;  Regulate the balance of osteoblasts and osteoclasts. | 18297083  33027692  12411305  32526405 |
| Heart | Play a vital role in anti-myogenicity and the generation of endocardial and endothelial cells;  Affect the remodeling and maturation of atrioventricular duct, as well as the whole process of EMT and heart valve formation. | 15466159  18071321,18497317 26635389 |
| Vasculature | Mediate the dynamic equilibrium transformation of endothelial cells to tip cells and stem cells through lateral inhibition;  Maintain arterial characterization in arteriovenous norms. | 28924218  17259972  33299176 |
| Hematopoietic system | Mediate hematopoietic stem cell self-renewal and maintenance of stemness;  Determine the lineage formation and differentiation of T cells;  Involved in the formation and regulation of subsets of B cells and innate lymphocytes. | 20207228  23665520  15146182  34453879 |
| Liver | Differentiation from hepatoblasts to bile duct cells;  Asymmetric development of the bile duct;  Transformation of liver cells, regeneration of biliary tract. | 20069650  23500150  34343491 |
| Nervous system | Promote the proliferation of neural stem cell;  Inhibit neuronal differentiation;  Participates in the regulation of neural crest cell differentiation. | 11101851  10706613  7893132 |
| Lung | Activate alveolar morphogenesis and maintains airway epithelial integrity;  Mediate the balance between proliferation and differentiation of basal cells. | 29025966  27364009  21624809 |
| Skin | Promote cell differentiation, while the NOTCH player in hair follicles inhibits cell differentiation, promotes proliferation, and maintains stemness. | 30143626  16618808  21703454 |
| Gastrointestinal tract | Inhibite secretory lineage differentiation of epithelial cells and promote the self-proliferation of stem cells. | 32878924  26933171  10476967 |
